# Single-cell multi-ome and immune profiles of the Inspiration4 crew reveal conserved, cell-type, and sex-specific responses to spaceflight

**DOI:** 10.1038/s41467-024-49211-2

**Published:** 2024-06-11

**Authors:** JangKeun Kim, Braden T. Tierney, Eliah G. Overbey, Ezequiel Dantas, Matias Fuentealba, Jiwoon Park, S. Anand Narayanan, Fei Wu, Deena Najjar, Christopher R. Chin, Cem Meydan, Conor Loy, Begum Mathyk, Remi Klotz, Veronica Ortiz, Khiem Nguyen, Krista A. Ryon, Namita Damle, Nadia Houerbi, Laura I. Patras, Nathan Schanzer, Gwyneth A. Hutchinson, Jonathan Foox, Chandrima Bhattacharya, Matthew Mackay, Evan E. Afshin, Jeremy Wain Hirschberg, Ashley S. Kleinman, Julian C. Schmidt, Caleb M. Schmidt, Michael A. Schmidt, Afshin Beheshti, Irina Matei, David Lyden, Sean Mullane, Amran Asadi, Joan S. Lenz, Omary Mzava, Min Yu, Saravanan Ganesan, Iwijn De Vlaminck, Ari M. Melnick, Darko Barisic, Daniel A. Winer, Sara R. Zwart, Brian E. Crucian, Scott M. Smith, Jaime Mateus, David Furman, Christopher E. Mason

**Affiliations:** 1 Department of Physiology and Biophysics, Weill Cornell Medicine, New York, NY 100221 USA; 2 The HRH Prince Alwaleed Bin Talal Bin Abdulaziz Alsaud Institute for Computational Biomedicine, Weill Cornell Medicine, New York, NY 10021 USA; 3 Center for STEM, University of Austin, Austin, TX USA; 4 BioAstra, Inc, New York, NY USA; 5 Division of Endocrinology, Department of Medicine, Weill Cornell Medicine, New York, NY 10065 USA; 6 Meyer Cancer Center, Weill Cornell Medicine, New York, NY 10065 USA; 7 Buck Artificial Intelligence Platform, Buck Institute for Research on Aging, Novato, CA 94945 USA; 8 Department of Health, Nutrition, and Food Sciences, Florida State University, Tallahassee, FL USA; 9 Tri-Institutional Biology and Medicine Program, Weill Cornell Medicine, New York, NY 10021 USA; 10 Division of Hematology/Oncology, Department of Medicine, Weill Cornell Medicine, New York, NY USA; 11 Cornell University, Meinig School of Biomedical Engineering, Ithaca, NY 14850 USA; 12 Department of Obstetrics and Gynecology, Division of Reproductive Endocrinology and Infertility, University of South Florida Morsani College of Medicine, Tampa, FL USA; 13 Department of Stem Cell Biology and Regenerative Medicine, Keck School of Medicine, University of Southern California, Los Angeles, CA USA; 14 Children’s Cancer and Blood Foundation Laboratories, Departments of Pediatrics, and Cell and Developmental Biology, Drukier Institute for Children’s Health, Meyer Cancer Center, Weill Cornell Medicine, New York, NY USA; 15 Department of Molecular Biology and Biotechnology, Center of Systems Biology, Biodiversity and Bioresources, Faculty of Biology and Geology, Babes-Bolyai University, Cluj-Napoca, Romania; 16 School of Medicine, New York Medical College, Valhalla, NY 10595 USA; 17 NASA Center for the Utilization of Biological Engineering in Space (CUBES), Berkeley, CA 94720 USA; 18 Department of Bioengineering, University of California, Berkeley, Berkeley, CA 94720 USA; 19 Department of Bioengineering and Therapeutic Sciences, University of California, San Francisco, San Francisco, CA 94158 USA; 20 Sovaris Aerospace, Boulder, CO USA; 21 Advanced Pattern Analysis & Human Performance Group, Boulder, CO USA; 22 Department of Systems Engineering, Colorado State University, Fort Collins, CO USA; 23 Stanley Center for Psychiatric Research, Broad Institute of MIT and Harvard, Cambridge, MA 02142 USA; 24 Blue Marble Space Institute of Science, Space Biosciences Division, NASA Ames Research Center, Moffett Field, CA 94035 USA; 25 Space Exploration Technologies Corporation (SpaceX), Hawthorne, CA USA; 26 Leonard Davis School of Gerontology, University of Southern California, Los Angeles, CA 90089 USA; 27 Department of Immunology, University of Toronto, Toronto, ON M5S 1A8 Canada; 28 Division of Cellular & Molecular Biology, Toronto General Hospital Research Institute (TGHRI), University Health Network, Toronto, ON M5G 1L7 Canada; 29 Department of Laboratory Medicine and Pathobiology, University of Toronto, Toronto, ON M5S 1A8 Canada; 30 University of Texas Medical Branch, 301 University Blvd, Galveston, TX 77555 USA; 31 Biomedical Research and Environmental Sciences Division, NASA Johnson Space Center, Human Health and Performance Directorate, 2101 NASA Parkway, Houston, TX 77058 USA; 32 Stanford 1000 Immunomes Project, Stanford School of Medicine, Stanford, CA 94306 USA; 33 Instituto de Investigaciones en Medicina Traslacional (IIMT), Universidad Austral, CONICET, Pilar, Argentina; 34 The Feil Family Brain and Mind Research Institute, Weill Cornell Medicine, New York, NY 10021 USA; 35 WorldQuant Initiative for Quantitative Prediction, Weill Cornell Medicine, New York, NY 10021 USA

**Keywords:** Epigenomics, Immunogenetics

## Abstract

Spaceflight induces an immune response in astronauts. To better characterize this effect, we generated single-cell, multi-ome, cell-free RNA (cfRNA), biochemical, and hematology data for the SpaceX Inspiration4 (I4) mission crew. We found that 18 cytokines/chemokines related to inflammation, aging, and muscle homeostasis changed after spaceflight. In I4 single-cell multi-omics data, we identified a “spaceflight signature” of gene expression characterized by enrichment in oxidative phosphorylation, UV response, immune function, and TCF21 pathways. We confirmed the presence of this signature in independent datasets, including the NASA Twins Study, the I4 skin spatial transcriptomics, and 817 NASA GeneLab mouse transcriptomes. Finally, we observed that (1) T cells showed an up-regulation of FOXP3, (2) MHC class I genes exhibited long-term suppression, and (3) infection-related immune pathways were associated with microbiome shifts. In summary, this study reveals conserved and distinct immune disruptions occurring and details a roadmap for potential countermeasures to preserve astronaut health.

## Abstract (web summary)

Multiple omics platforms and deep single-cell profiling in the I4 astronauts reveal both conserved and distinct immune system disruptions across missions, provide a single-cell immune reference for future missions.

## Introduction

Human spaceflight exposes individuals and their immune systems to unique environmental factors, including microgravity, fluid shifts, and radiation^1,2^. Since 1961, over 675 astronauts have traveled to space; studies on some crew members have shown significant immune stress related to spaceflight^3^. Given that more individuals with diverse physical and biomedical backgrounds are traveling into space—and that many more commercial missions are planned (e.g., Polaris Dawn, Axiom)—an urgent goal of aerospace medicine is to understand better how the immune system responds to and recovers from spaceflight for the broader civilian population. Moreover, such profiles of cellular and molecular changes in commercial crews can guide personalized countermeasures for the missions and eventually lead to better crew performance and safety^4^.

Immune-related clinical symptoms from spaceflight are prevalent among astronauts and span many phenotypes, including inflammation, infection, and viral reactivation^5,6^. Indeed, clinical symptoms associated with immune dysregulation were reported with 3.4 events per flight year, representing 46% of crew members^6^. While these findings show that the spaceflight environment affects the human immune system, they do not reveal the underlying pathways and mechanisms therein, underscoring the need for further studies. Also, since spaceflight phenocopies many of the effects of aging and aging-related disease (e.g., bone density and muscle loss)^7^, a better characterization of immune system changes during the extreme physiological stressor of spaceflight can offer insights into immune dysregulation and functional deterioration on Earth.

Multi-omic studies provide unique advantages for understanding biological changes, including spaceflight-associated immune system alterations. For example, the NASA Twins Study used gene expression to investigate immune changes stemming from a year-long mission, and found unprecedented levels of cytokines like IL-6 and IL-10, and gene expression changes in both B-cells and T-cells^7–9^. However, a major limitation of that study was the sample size, as it involved only one flight subject. It additionally used a bulk-cell analysis for most of the transcriptome (preventing observations at a single-cell resolution) and lacked a chromatin analysis using assays for transposase-accessible chromatin accessibility (ATAC) data^8,9^, which is critical for understanding changes in genome regulation and long-term stress response.

Thus, a high-resolution map of the immune system’s response to spaceflight is still needed. With these needs in mind, we report here findings on the SpaceX Inspiration4 (I4) mission, an all civilian-crewed commercial orbital spaceflight, including multi-omic, in-depth immune system profiling at the single-cell level for the four-member crew with a broad age range (29-50 years old at launch) and biomedical backgrounds. We longitudinally analyzed single-nucleus gene expression (snRNA-seq), chromatin accessibility (snATAC-seq), and single-cell T/B cell antigen receptor sequences from peripheral blood mononuclear cells (PBMCs). In addition, we integrated this multi-omics data with a clinical profile of a complete blood count (CBC), a comprehensive metabolic panel (CMP), and cytokine/chemokine/growth factor/metabolite measures. We also profiled cell frequencies by fluorescence-activated cell sorting (FACS), biochemical profiling, differential gene/peak expression, enriched biological pathways, over-represented transcription factor binding site (TFBS), antibody isotype, mutation profiles of BCR/TCR, cell-free RNA (cfRNA) in plasma, and association with skin, oral, and nasal microbiome changes. Here, we provide a detailed report of these findings, illustrating a unique set of immune system changes induced by high-elevation spaceflight, and conclude with potential countermeasures derived from these data, all of which can guide and inform future studies and missions.

## Results

### Immune-metabolic changes after spaceflight and recovery

**Fig. 1:**
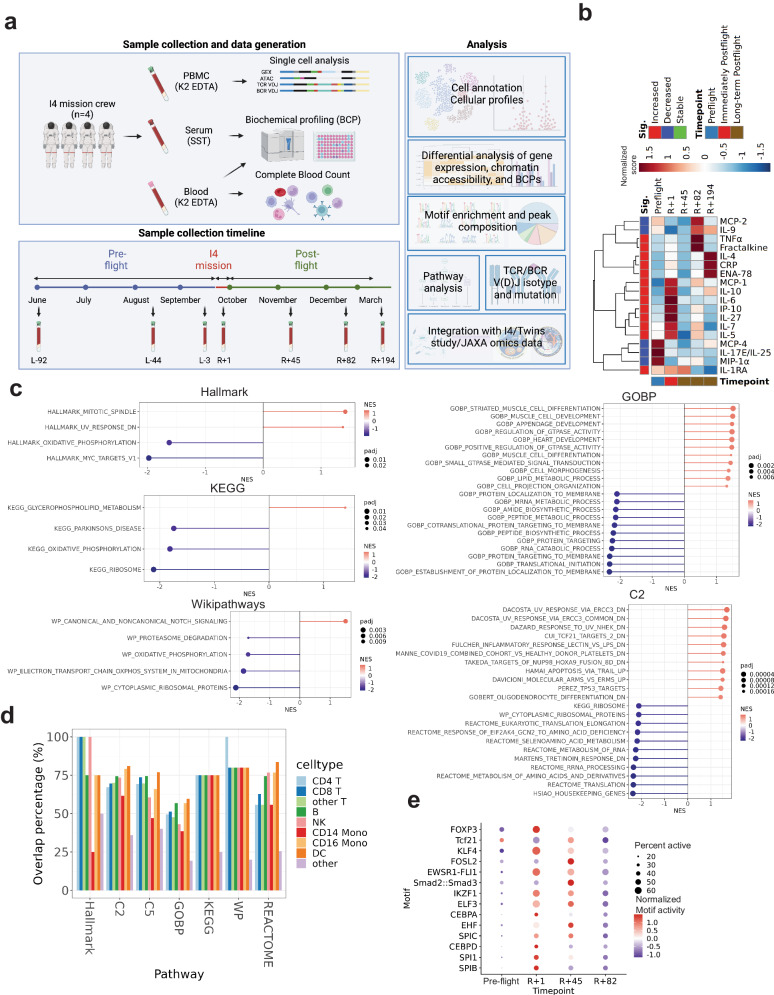
Immune-metabolic changes after 3-day spaceflight and recovery. **a** Overview of I4 mission single cell GEX + ATAC, single cell TCR/BCR V(D)J repertoire, biochemical profiles (BCP) of 97 analytes, and complete blood count (CBC) of 15 analytes data collection and analysis, created with BioRender.com. **b** Heatmap of significantly changed biochemicals (cytokines, chemokines, and growth factors) in serum before spaceflight (Pre-flight: mean of L-92, L-44, L-3) and after spaceflight (Immediately Post-flight: *R* + 1, and Long-term Post-flight: *R* + 45, *R* + 82, *R* + 194). A significant increase in concentration is observed immediately after spaceflight (*R* + 1) in IL-1RA, IL-4, IL-5, IL-6, IL-7, IL-10, IL-27, MCP-1, TNFα, IP-10, ENA-78, CRP and Fractalkine. On the other hand, IL-9, IL-17E/IL-25, MIP-1α, MCP-2 and MCP-4 showed a significant decrease in their serum levels after spaceflight (*R* + 1). Wilcoxon-rank sum test (padj <0.05, two-sided). **c** GSEA of the ‘spaceflight signatures of the I4 astronauts’ (Hallmark, KEGG, and wikipathways: filtered with padj <0.05, GOBP and C2: top10 of positive and negative NES, padj < 0.05, padj calculated by fGSEA R package). **d** Overlap percentage of the GSEA pathways across the I4 immune cells (Fisher’s exact test, two-sided, padj < 0.05. Except for the Hallmark CD14 Mono: *P* value = 0.09). **e** Activity scores of top enriched motifs from pseudo-bulk PBMCs over time. Source data are provided as a Source Data file.

To characterize the immune and metabolic changes induced by spaceflight, we collected serum and whole blood from the crew before and after the spaceflight (Fig. 1a) and first performed CBC and CMP assays to measure cytokines, chemokines, growth factors, and metabolites. The crew’s baseline values (pre-flight) were compared to those obtained 24 h after returning to Earth (*R* + 1) and post-flight (*R* + 45, 82, 194), which revealed a set of cytokines with significant increase (*n* = 13) (Fig. 1b, Supplementary Fig. 1a, b, Wilcoxon-rank sum test, adjusted *p* value < 0.05) and a smaller set (*n* = 5) of molecules with a significant decrease (Supplementary Fig. 1a, b, Wilcoxon-rank sum test, adjusted *p* value < 0.05). The IL-6, IL-10, CRP, and MCP-1 increases were consistent with changes observed in other astronauts following long duration (3–6-month or 12-month duration) missions (Supplementary Fig. 1c)^7–10^. Moreover, several other pro-inflammatory cytokines (TNFα, IL-27, CRP) and chemokines (IP-10, ENA-78, Fractalkine) were also significantly up-regulated at *R* + 1 (Fig. 1b). Although IL-6 and TNFα are well-known pro-inflammatory cytokines, acute phase reactants like fibrinogen, hemoglobin, and SAP levels did not significantly change (Supplementary Fig. 1b), indicating that the immune reaction was not pervasive, and no other significant changes were observed in the basic metabolic blood panel (Supplementary Fig. 1d).

These data suggest that a high elevation, 3-day spaceflight is sufficient to induce the production of some established spaceflight cytokine signatures (e.g., IL-6, IL-10) as well as previously undocumented, responsive cytokines (e.g., ENA-78). These proteins have critical roles in immune response, muscle homeostasis, and hematopoiesis^8^, but are not normally associated with systemic inflammation (Supplementary Fig. 1e). Moreover, some cytokines (e.g., IL-6, IL-7, IL-10, IL-1RA, and Fractalkine) are considered exerkines, cytokines produced by the muscle and other tissues during exercise. As such, we then examined if muscle tissues could be the source of these immune markers. Significant increases in some myokines (IL-4, IL-5, and IL-7) were indeed observed, further supporting muscles as a possible source (Fig. 1b) of these molecules, and indicating a physiological response to microgravity rather than a purely inflammatory response^8^.

We next mined RNA-sequencing data from dissected, space-flown mouse tissues to assess a possible tissue-of-origin for the circulating cytokines (Supplementary Fig. 2a). While non-muscle tissues (e.g., mandibular bone, brown and white adipose tissues) did not show changes in IL-6 or IL-10, (Supplementary Fig. 2b, d), the soleus showed a significant increase in the chemokine Ccl2/Mcp-1 (padj < 0.05, logFC > 1), which is a chemokine associated with muscle exertion. These samples also showed high CD68 expression, a surface marker highly expressed in differentiating (M0) macrophages (Supplementary Fig. 2e–h). Also, the tibialis anterior muscle showed an increase in interleukins, with the largest increase upon landing for the pro-inflammatory cytokine IL-5 (log2FC = 2.3, *p* value < 0.05) (Supplementary Fig. 2i), further suggesting muscles as a potential source for the cytokines found in the I4 crew.

### A spaceflight gene expression signature in Inspiration4 astronauts

**Fig. 2:**
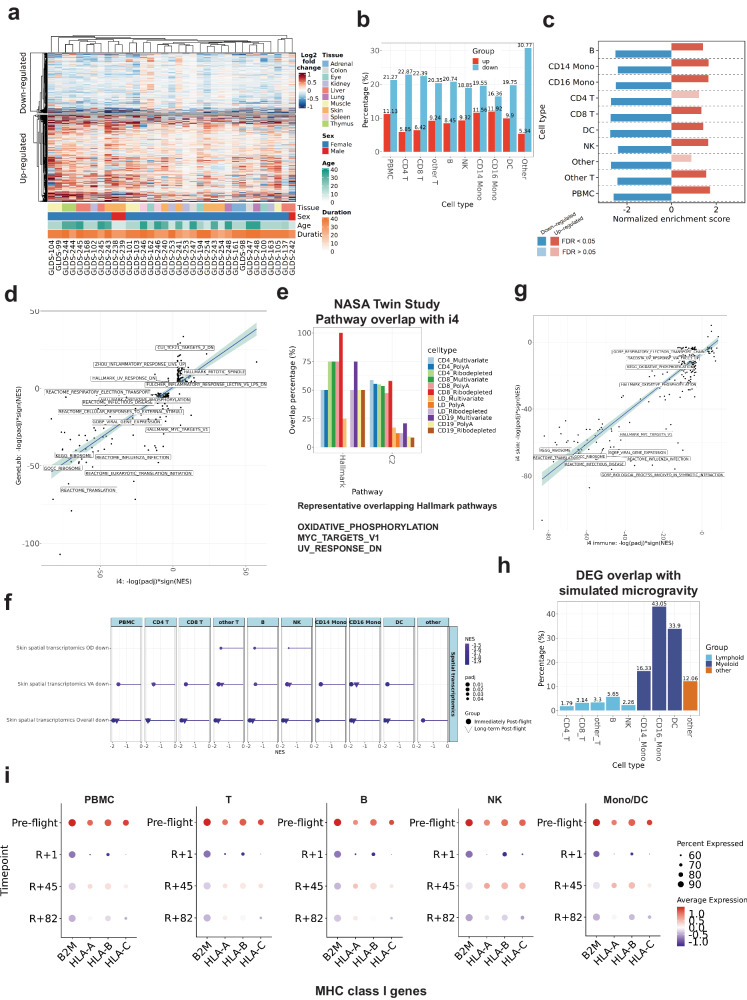
Conserved and distinct spaceflight signatures across mission, species and mission duration. **a** Log2 fold change heatmap of the “spaceflight signatures in mice” in 27 datasets with 10 different mouse tissues. Age (Day). Duration (Day). 1,288 up-regulated and 896 down-regulated genes. **b** The up-regulated genes (red) and down-regulated genes (blue) from the I4 data are shown in terms of percentage of overlap (y-axis) with the ‘spaceflight signatures in mice’. **c** GSEA analysis the I4 DEGs with the ‘spaceflight signatures in mice’. **d** Scatter plot of the -log10(padj)*sign(NES) of the ‘spaceflight signatures of the I4 astronauts’ and the ‘spaceflight signatures of mice’ GSEA pathways and the representative pathways. Pearson correlation (R) = 0.82. Slope: 0.69. Two-sided. The standard error should be used to create the band around the linear regression line. **e** Overlap percentage of the significantly enriched overlapped GSEA pathways (NASA Twins vs I4). **f** GSEA of PBMC and subpopulations at the immediately post-flight (R + 1) and long-term post-flights (R + 45 and R + 82) with up-regulated and down-regulated DEGs of skin spatial transcriptomics data (padj <0.05). OE Outer Epidermis, OD Outer Dermis, VA Vasculature) in skin biopsy data. The fgsea analysis employs a one-sided permutation-based test to determine the significance of gene set enrichment, with raw *p* values adjusted for multiple testing using the Benjamini-Hochberg procedure to control the false discovery rate (FDR). **g** Scatter plot of the −log10(padj)*sign(NES) of the ‘spaceflight signatures of the I4 astronauts’ and the I4 skin spatial transcriptomics GSEA pathways and the representative pathways. Pearson correlation (*R*) = 0.87. Slope: 0.85. Two-sided. The standard error should be used to create the band around the linear regression line. **h** The percentage of overlap of I4 DEGs and in vitro microgravity simulated DEGs. **i** MHC class I gene expression in the I4 immune cells. Source data are provided as a Source Data file.

PBMCs were used to generate single-nuclei gene expression (GEX) and ATAC data (Fig. 1a, b, Fig. 2). We combined GEX and ATAC analysis of PBMCs across 151,411 nuclei (filtered with minimum of 200 genes, maximum of 4500 gene counts, maximum of 20% mitochondrial reads, maximum of 100,000 peak counts, maximum of 2 nucleosome signal, and minimum of TSS enrichment per cell) by adapting the multi-omic pipeline reported^11^, spanning 9 immune cell types: CD4 T cells, CD8 T cells, other T cells, B cells, natural killer (NK) cells, CD14 monocytes, CD16 monocytes, dendritic cells (DCs), and all remaining cells (“other”) (Supplementary Fig. 3a). We validated the expression of markers specific for each subpopulation in annotated PBMC subpopulations based on reference expression for each cell type (Supplementary Fig. 3a), quantified the distribution of cell populations (Supplementary Fig. 3b) and confirmed that overall cell proportions were stable across spaceflight in the combined single-nuclei multi-ome and FACS analysis (Supplementary Fig. 3c–e) and CBC data (Supplementary Fig. 3f), and mapped principal component analysis (PCA) clustering of GEX and ATAC data across the timepoints (Supplementary Fig. 4, 5).

Next, differentially expressed genes (DEGs) and differentially accessible regions (DARs) (padj <0.05, |log2FC | > 0.25) were calculated from the snRNA-seq snATAC-seq data, respectively, comparing immediate (R + 1) and post-flight differences (*R* + 45, *R* + 82) relative to pre-flight levels (Supplementary Fig. 6a). The number of DEGs and DARs were highest at R + 1, but decreased for subsequent timepoints among PBMCs, T cells, B cells, NK cells, and monocytes (Supplementary Fig. 6b). However, a wide range of responses to spaceflight was observed, with some cell types more affected than others. For example, CD14 monocytes showed the highest number of DARs and DEGs at R + 1, yet CD4 T and CD8 T cells were the least perturbed (Supplementary Fig. 6b–c). Notably, chromatin accessibility responses to spaceflight exhibited even greater cell type-specificity (Supplementary Fig. 6b, c) than DEGs, with the most changes still appearing at *R* + 1. In addition to the cell-specific responses to spaceflight, a core set of pan-cellular gene expression changes was observed at *R* + 1. Specifically, a set of 144 consistent DEGs was observed across all nine cell types (Supplementary Fig. 6c).

PBMCs DEGs were significantly conserved in I4 immune cells (Supplementary Fig. 6d), thus, we used the PBMCs DEGs as the “spaceflight signatures of the I4 astronauts” for further analysis. Given this spaceflight signature set of genes, we then examined the enriched pathways using Gene Set Enrichment Analysis (GSEA), which showed enrichment of ribosomal translation, oxidative phosphorylation (OXPHOS), mitochondrial metabolism, UV response, TCF21 targets, mitotic spindle, and immune pathways (padj < 0.05) (Fig. 1c). Additionally, we confirmed that the GSEA result was conserved across all I4 immune cell sub-types (Fig. 1d). We cross-validated the GSEA findings with over-representation analysis through Gene Ontology pathways of these DEGs, which also showed down-regulated DEGs were enriched (padj < 0.05) in ribosomal translation, oxidative phosphorylation, and mitochondrial metabolism (Supplementary Data. 1). Up-regulated DEGs were significantly enriched in response to stimulus, signaling, and metabolism (Supplementary Data. 1). Moreover, Ingenuity Pathway Analysis (IPA) also showed inhibition of oxidative phosphorylation and translation (EIF2 signaling) pathways and activation of mitochondrial dysfunction across cell types (Supplementary Data 2). Also, the core set of altered regions of chromatin (DARs) on R + 1 included over-represented motifs for SPIB, SPI1, CEBPD, SPIC, EHF, CEBPA, ELF3, IKZF1, EWSR1-FLI1, FOSL2, and KLF4 (Fig. 1e, Supplementary Fig. 6e, Supplementary Data 3). Their normalized motif activity score (chromVAR deviation z-scores of TF motifs) were increased at *R* + 1 and recovered over time (Fig. 1e). Of note, while CEBPA^12^, EHF^13^, SMAD^14^, EWSR1-FLI1^15^, c-Fos^16^, and TCF21^17,18^ have been implicated in spaceflight before, the others have not been previously described in astronauts.

### Cell subpopulation changes after 3-day spaceflight and recovery

To better understand spaceflight’s impact on each cell type, we examined the cell profiles with more granular sub-typing (Supplementary Fig. 7a–d), mutation rates in B cells and T cells (Supplementary Fig. 7e–h) and used single-sample GSEA (ssGSEA) on the DEGs for each immune cell population (Supplementary Fig. 8, Supplementary Data 4). First, overall cell proportions of granular annotation were stable across spaceflight (Supplementary Fig. 7a–d). Second, we found that the TCR V(D)J repertoire from L-3 to R + 82 was stable in terms of mutations (Supplementary Fig. 7e, f). Conversely, the total number of BCR mutations increases relative to pre-flight at *R* + 1 (64.6 vs 61.6) and *R* + 45 (65.5 vs 61.6) (Supplementary Fig. 7g, h). Moreover, we found that cell differentiation and cell proliferation pathway scores were decreased (around 5–10%) at *R* + 1 for both CD4 T and CD8 T cells, which mirrors the T cell suppression previously observed in simulated GCR irradiation^19^, simulated microgravity^20^, and Space Shuttle missions^21^ (Supplementary Data 4). Similarly, the CD4 central memory T cell (CD4 TCM) differentiation pathway and memory T cell activation scores were decreased in CD4 effector memory T cells (CD4 TEM) and CD8 central memory T cells (CD8 TCM) at *R* + 1. However, monocytes and DCs showed more mixed phenotypes, with activation, differentiation, aggregation, and extravasation scores all dysregulated at *R* + 1. These findings are consistent with previous reports of changes in phagocytic capacity, degranulation, and cytokine production for DC^22,23^ (Supplementary Fig. 8).

Upon return to Earth, the astronauts’ serum showed higher concentrations of cytokines associated with inflammation, such as IL-6, TNF-α, APR (acute phase reactants), and C-Reactive Protein (CRP) (Fig. 1b). These changes were concomitant with an increase in cytokines with anti-inflammatory activity, such as IL-1ra, IL-10, and cytokines linked to a Th2 profile (IL-4 and IL-5) (Fig. 1b), which likely balances the inflammation from returning to Earth. However, the Th1-, Th2-, and Th17-secreted cytokines in CD4 TCM subset^24–28^ showed no significant changes (Supplementary Fig. 9a); GSEA analysis of Th1, Th2, and Th17 cell pathways confirmed no significant changes (Supplementary Fig. 9b, padj > 0.05), further indicating the cell-type specificity of these responses. Interestingly, significant changes were also observed in chemokines related to the migration of monocytes (increased MCP-1, decreased MIP-1α, and MCP-2/4) and neutrophils (higher IP-10 and ENA-78) (Fig. 1b).

To examine the cellular impact of these cytokine changes, we mapped known vs. novel pathways associated with spaceflight^7^, as well as TF accessibility and the cell-type specificity of these responses. Of note, the CD16 monocytes showed the highest number of enriched, known pathways (Supplementary Fig. 9c), and CD14 monocytes showed the longest persistence of these changes, indicating a slower recovery after spaceflight. We then used the DARs’ chromatin accessibility data to compare over-represented TF motifs at R + 1 (Supplementary Data 3), and intersected these TFs with the literature. For the top 12 motifs from T cells, a novel set of spaceflight-responsive TFs was identified (KFL16, ETV1, ZNF148, RREB1, GABPA, KLF9), as well as some known, including MAZ^29^, Wt1^30,31^, KLF4^13^, and KLF5^32^. For B cells, novel TFs included SPIB, ZKSCAN5, IKZF1, and EBF3, while previously identified TFs included STAT^33^, IRF1^34^, EHF^13^, and Arid3a^18^. Interestingly, these TFs are all key to B cell function: STATs mediate cytokine responses (IL7^35^, B cell differentiation, and control of the germinal center reaction^36,37^); SPIB regulates the expression of the B cell receptor, CD40L, BAFF, and TLR ligands^38^, and IKZF1 is a transcription factor essential for B cell activation, maturation, and differentiation^39^. For monocytes and DCs, novel TFs included SPIB, SPIC, HLF, IKZF1, ELF3, CTCFL, and ZKSCAN5, and known TFs included CEBPD^40^, STAT1^40^, CTCF^41^, EHF^13^, Arid3a^18^, and IRF1^34^. Finally, we observed that the motif activity scores of CD14 monocytes and DCs recovered more slowly than CD16 monocytes, taking until the last time point to recover (Supplementary Fig. 9d), further evidence that the CD14 population was the slowest to recover from spaceflight.

### Conserved responses from other missions and possible countermeasures

To further validate and contextualize the I4 spaceflight signature, we next compared differential genes and pathways to several other data sets, including gene expression data in NASA’s GeneLab database, the NASA Twins Study^7^, the I4 skin spatial transcriptomomes^42^, I4 plasma cfRNA, I4 EVP and plasma proteomics^43^, the JAXA cfRNA study, and microgravity-simulated PBMCs^44^. First, we analyzed 27 murine datasets from GeneLab (*n* = 817 samples) for spaceflight-specific DEGs, and found 2184 total DEGs (1288 upregulated and 896 downregulated) (Fig. 2a) common across the 27 datasets (Supplementary Data 5). When comparing orthologous genes to the human I4 data, we found overlapping DEGs for both upregulated (5–12%) and downregulated (16–30%) genes (Fig. 2b). Moreover, compared to the human data, the “murine spaceflight signature” was directionally consistent in all comparisons (20/20, 100%) and statistically significant (18/20 pathways, 90%) by GSEA for both up and down-regulated genes (padj < 0.05) (Fig. 2c) and Fisher’s exact test (padj < 0.05) (Supplementary Fig. 10a), indicating a core set of human and murine responses to spaceflight.

We next expanded the human and mouse comparisons at the pathway level. Over-representation analysis of the murine spaceflight signature showed enrichment of ribosomal translation and metabolism in down-regulated DEGs and signaling, response to stimulus, and metabolism in upregulated DEGs, overlapping with the I4 pathways (Supplementary Data 1 and 6). The GSEA result of the I4 data and the murine spaceflight signature showed statistically significant overlap (Fisher’s exact test. padj; Hallmark: 3.845e-02, C2: 3.317e-47, C5: 2.341e-83) (Supplementary Fig. 10b). Down-regulated pathways consistent across both cohorts included OXPHOS, Myc targets, ribosome, infectious disease, viral gene expression, influenza infection, and translation pathways (Supplementary Fig. 10b). Also, mitotic spindle, UV response DN pathway, and TCF21 targets pathway were also consistently, significantly up-regulated (Supplementary Fig. 10b). Of note, the pathway scores (−log10(padj)*sign(NES)) were positively correlated in the I4 murine signatures (Pearson correlation, *R* = 0.82, slope: 0.69) (Fig. 2d). Next, we compared the I4 data to the NASA Twins study immune cell RNA-seq^7–9^, and found that the immune cell DEGs were statistically significantly for up- and down-regulated DEGs when compared to the I4 data (Supplementary Fig. 10c). Additionally, the Twins’ GSEA profiles overlapped with the I4 GSEA profiles, showing enrichment in OXPHOS, Myc targets v1, and UV response, consistent with the I4 signature (Fig. 2e). These results were confirmed in the I4 skin spatial transcriptome data, where the skin and subregion down-regulated DEGs were statistically significantly (padj < 0.05) down-regulated in I4 data (Fig. 2f) and the pathway scores were positively correlated in the I4 (Fig. 2g), including ribosomal translation, OXPHOS, UV response pathways (padj < 0.05) (Supplementary Fig. 10d) in response to spaceflight. However, EVP and plasma proteomics data^45^ (Supplementary Fig 10e, padj < 0.05, GSEA) were distinct from the OXPHOS pathways, but did overlap with NPM1, immunoglobulin complex, and blood microparticle pathways.

Changes in gravity and radiation both occur in spaceflight, but are confounded in most studies, making it difficult to delineate which factor leads to the cellular changes observed in astronauts. To better distinguish between the contributions of radiation and microgravity, we compared the I4 data to a set of 375 DEGs observed in single-cell RNA-seq data from in vitro PBMCs exposed to simulated microgravity (μG) for 25 h. Monocytes (e.g., CD14) and dendritic cells, which are myeloid-derived cells, showed higher DEG overlap (16–43%) vs. the lymphoid-derived cells (2–5% for T cells, B cells, and NK cells), giving further evidence that lymphoid-derived cells are less sensitive to microgravity (Fig. 2h, Supplementary Fig. 10f). GSEA analysis of μG-simulated DEGs showed no significant enrichment in OXPHOS, ribosomal translation, and UV response (Supplementary Fig. 10g), indicating that most of the responses in PBMCs are likely from radiation and other spaceflight stressors, rather than changes in gravity.

Since the expression of cluster of differentiation (CD) and Human leukocyte antigens (HLA) markers is known to change after spaceflight^46^, we further analyzed the expression of CDs and HLAs in our immune cells. We found the long-term suppression of MHC class I genes (HLA-A, HLA-B, HLA-C, B2M) in the I4 immune cells (Fig. 2h). This long-term suppression was cross-validated in the NASA Twins Study data, the JAXA cfRNA profiles, and the I4 plasma cfRNA, which showed sometimes a spike in activity in flight, but a consistent and significant (*q* value < 0.05, FDR-corrected) decrease of MHC class I genes (HLA-A, HLA-B, HLA-C, B2M) genes post-flight across all missions and most cell types (Supplementary Fig. 11).

Finally, to identify potential drugs or supplements that can reverse these effects on the immune system, we used a novel compound-gene interactome machine learning algorithm to identify drugs and vitamins that significantly map to altered genes^44^. Using these algorithms, we identified 148 compounds that significantly map to DEGs of PBMCs and subpopulations (Supplementary Data 7). Based on PBMC subpopulation alterations, these potential compounds could identify biological targets for countermeasure development to optimize human performance and could be tested to mitigate potential negative health effects resulting from spaceflight exposure^2,47^.

### Spaceflight-induced immune dysregulation mirrors monocyte dysregulation, infection phenotypes, TCF21, and FOXP3 regulation

**Fig. 3:**
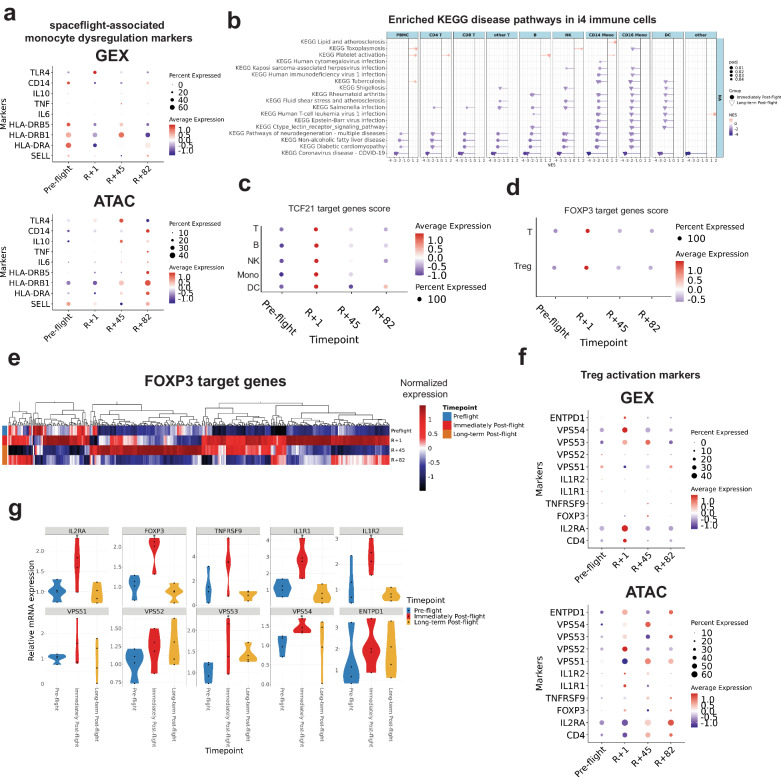
Immune cell sub-population changes after spaceflight and recovery profiles. **a** Dot plots of previously reported spaceflight-associated CD14 monocyte markers (Top: gene expression, Bottom: ATAC derived gene expression). **b** GSEA of PBMC and subpopulations with the selected KEGG pathway significantly enriched with the over-representation analysis of the I4 immune cell DEGs (padj < 0.05). The fgsea analysis employs a one-sided permutation-based test to determine the significance of gene set enrichment, with raw p-values adjusted for multiple testing using the Benjamini-Hochberg procedure to control the false discovery rate (FDR). **c** Activity scores of TCF21 target genes in T, B, NK, monocyte, and dendritic cells. **d** Activity scores of FOXP3 target genes in T and Treg cells. **e** Heatmap of FOXP3 target genes in Treg cells. Color scale represents the normalized expression. **f** Dot plot of Treg markers and Treg activation markers in Treg cells (Left: gene expression. Right: ATAC derived gene expression). **g** Relative mRNA expression of Treg markers and Treg activation markers in T cells quantified by qPCR. Source data are provided as a Source Data file.

The prolonged dysregulation of CD14 monocytes observed in our data (Supplementary Fig. 9c) led us to next examine immune-associated clinical symptoms reported with spaceflight^6,48^, especially since monocytes are responsible for cytokine production, phagocytosis, and antigen presentation. First, to validate the CD14 dysregulation, we compared the I4 CD14 monocyte data to previously reported monocyte dysregulation markers linked to clinical symptoms^22,23,49^, which confirmed the down-regulation of five known markers in the I4 CD14 monocytes (e.g., SELL, HLA-DRA, HLA-DRB5, IL6, CD14) and an increase in TLR4 across both gene expression and ATAC-seq data (Fig. 3a). When examining I4 immune cell DEGs (padj < 0.05) (Supplementary Data 8), we also found a decrease in the pathways associated with response to infection, including COVID-19, Kaposi sarcoma-associated herpesvirus, CMV, tuberculosis, graft-versus-host defense, and *Salmonella* infection (KEGG pathways, p-adj < 0.05, Supplementary Data 8), and validated with a GSEA analysis (Fig. 3b). Finally, to examine the capacity for B-cell function, we examined the increased BCR mutation rate (Supplementary Fig. 7g), which is caused by somatic hypermutation secondary to antigen exposure or inflammatory stimuli. Indeed, single-cell expression data suggested possible up-regulation of BCR signaling pathway genes in B cells immediately post-flight (Supplementary Fig. 12a).

Given the TCF21 and FOXP3 pathway changes (Fig. 1c, Fig. 2d, Supplementary Fig. 12b, c), we next examined the activity of target genes in cellular subtypes. For all immune cells, the TCF21 target gene enrichment score was increased at *R* + 1 (Fig. 3c). And it is confirmed by the motif activity score of ATAC data in T and B cells (Supplementary Fig. 9d). In T cells, FOXP3 target gene enrichment score was increased (Fig. 3d), and again confirmed by the motif activity score of ATAC data (Supplementary Fig. 9d). Changes in FOXP3 suggested changes in T cell regulation during spaceflight^50–52^, and potentially altered immune function post-flight, which is in line with the GSEA results and KEGG pathways (Fig. 3b, Supplementary Data 8). We further validated FOXP3 upregulation in T cells and Treg cell activation after spaceflight by analyzing Treg activation markers^53^ and FOXP3 target genes across T cells (Fig. 3e, f, Supplementary Fig. 12d). Our data indeed showed an increase in Treg activation markers at *R* + 1, which recovered over time in both gene expression and ATAC data in both T cells and Treg cells (Fig. 3f, Supplementary Fig. 12d). Finally, we orthogonally validated the FOXP3 expression and Treg activation markers^53^ in FACS-isolated T cells by qPCR, which showed a post-flight increase in expression at *R* + 1 in all targets (Fig. 3g), and a statistically significant increase (Student’s *t* test, padj < 0.1) for FOXP3, IL1R1, IL1R2, IL2RA, VPS53, and VPS54.

### Sex-dependent differences in response to spaceflight and recovery

**Fig. 4:**
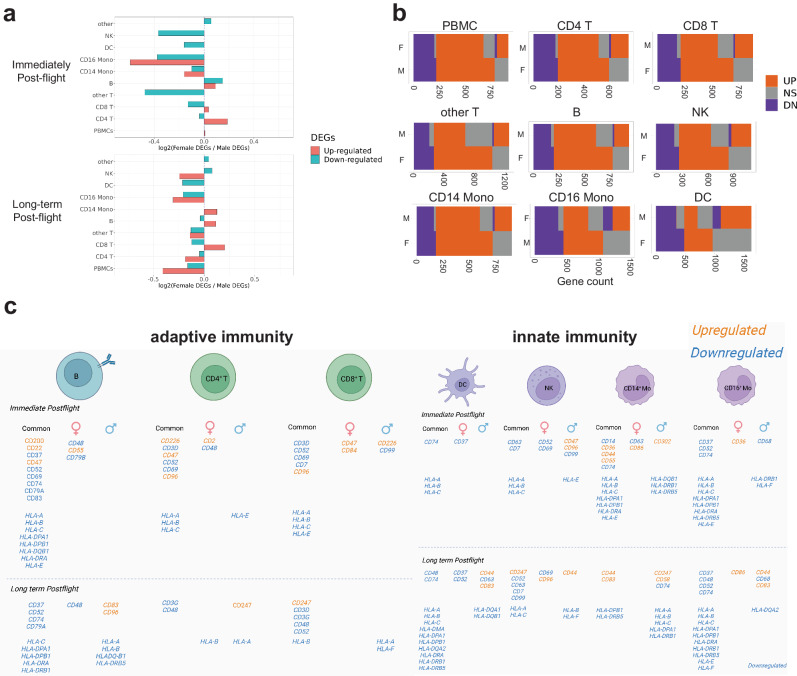
Sex-dependent differences in response to spaceflight and recovery. **a** Log2 Female to male DEGs ratio immediately post-flight (Top) and long-term post-flight (Bottom). The dotted line represents a Log2 ratio of 0. **b** Heatmap plot represents the overlap of up-regulated DEGs (Orange) and down-regulated DEGs (Purple) from females and males of PBMC and subpopulations. F Females, M Males. **c** Common and sex-specific HLA and CD expressions in immune cells, created with BioRender.com. Source data are provided as a Source Data file.

Since little is known about sex-dependent differences in response to spaceflight^54,55^, we next examined differential effects on the immune systems of the I4 crew by sex. First, we compared the ratio of up- and down-regulated DEGs (Fig. 4a) between males and females, which showed a higher number of DEGs in males for almost all cell types. Second, the overlap of up-/down-regulated DEGs between females and males showed a partial overlap (Fig. 4b), with down-regulated DEGs showing a higher overlap percentage (average of up-regulated genes: 51.9%, average of down-regulated genes: 84.1%). Next, GSEA was performed to gain a pathway-based view of sex-dependent gene expression changes. Down-regulated pathways for OXPHOS and Myc targets and up-regulated pathways for mitotic spindle and UV response were conserved between the sexes (Supplementary Fig. 13a). To expand upon these results, we used Ingenuity IPA for the sex-specific DEGs (padj < 0.05). More pathways were altered in males at R + 1 across almost all cell types (Supplementary Data 9). Notably, gene expression differences in estrogen signaling have also been observed in female vs. male B cells^56^.

We further analyzed the CD and HLA genes with sex-specific or common, DEGs in the immune cells (Fig. 4c, Supplementary Data 10). Both CD79A and CD79B were down-regulated (adj-*p* value < 0.01) in females (*R* + 1), and other CDs also showed negative or positive female-to-male (F/M) log2FC ratios (Fig. 4c). In B cells, CD69 and CD83 showed down-regulation at landing for both sexes, yet CD55 was upregulated only in females. HLA-A, HLA-B, and HLA-C were all down-regulated in all immune cells in both sexes, immediately post-flight (Fig. 4c), but only males showed persistent HLA-A downregulation post-flight. In DCs, NK cells, and CD4 T cells, males showed down-regulation of ribosomal protein-encoding genes and OXPHOS (Supplementary Data 9). OXPHOS was inhibited in female B cells, and mitochondrial genes were also downregulated only in females. The CD4 and CD8 DEGs showed activation of T cell receptor (TCR) signaling (z-scores: 2 and 2.449, respectively) at *R* + 1 in females (Supplementary Data 9). In contrast, TCR signaling was inhibited, with unique DEGs in male DCs. There was also a significant up-regulation (Wilcoxon rank sum test, *p* value < 0.01) of HLA-DQB1 F/M ratio in DCs (Supplementary Data 10).

Leveraging the ATAC-seq data, we compared the male/female activity scores of enriched DNA motifs at *R* + 1 for all cell types. In CD4 and CD8 T cells, the motif activity scores at *R* + 1 were higher in females, but the recovery pattern was similar (Supplementary Fig. 13b, Supplementary Data 11). In B cells and CD16 monocytes, the motif activity scores were higher in females at *R* + 1 but higher in males at *R* + 45, indicating these chromatin changes persisted longer in males. In NK cells and other cell types, the motif activity scores were higher in males at *R* + 1. Additionally, the activity scores of CEBPB, CEBPD, and CEBPE were higher in males at *R* + 45 in CD14 monocytes, further pointing to CD14’s significance and long-term chromatin disruption.

Finally, to discern the generalizability of sex-specific changes, we used a replication cohort of 64 NASA astronauts and compared it to the I4 mission, and we also examined sex-dependent changes in serum BCPs from a study with 27 other targets^57^ (Supplementary Data 12). These additional data showed that cytokine and acute phase reactant proteins (i.e., IL-8, CRP, and fibrinogen) confirmed differences across sex and mission length. In both males and females, mean serum CRP levels were higher on R + 0 compared to L-180 (2.1 ± 2.4 vs 1.3 ± 1.9 mg/L and 3.7 ± 5.7 vs 1.7 ± 2.2 mg/L, respectively), and mean CRP values were higher in females than males (Supplementary Data 12). Sex and time interaction was observed in IL-6, IL-8, and fibrinogen, and there was a significant difference between males and females for IL-8 and fibrinogen (Post hoc Bonferroni t-test, *q* value < 0.05).

### Spaceflight-associated immune cell gene expression changes potentially associated with microbiome abundance

**Fig. 5:**
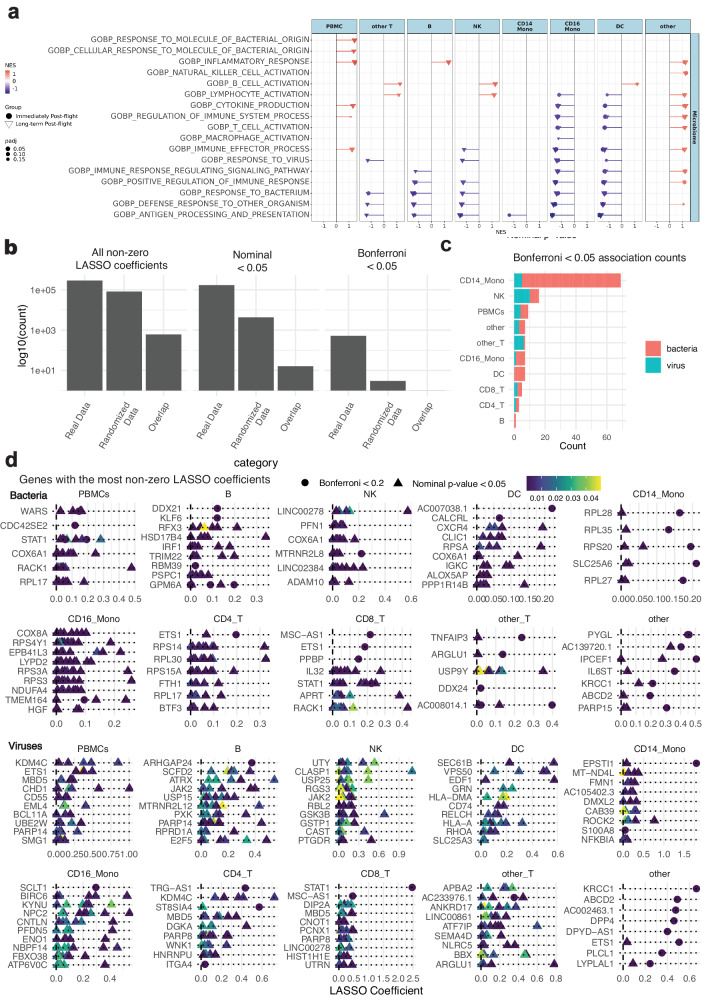
The landscape of microbiome-immune associations. **a** GSEA of all immune cells with the significantly enriched GO-BP pathways from the microbiome-associated immune cell DEGs related to immune function (padj <0.2). The fgsea analysis employs a one-sided permutation-based test to determine the significance of gene set enrichment, with raw p-values adjusted for multiple testing using the Benjamini-Hochberg procedure to control the false discovery rate (FDR). **b** We compared the associations reported in the main text to associations run on randomized data, computing the overlap therein at different stringency levels for controlling false positives. The three bars in each sub-panel correspond to the number of associations in the “real” (log-transformed) data versus randomized data and the overlap therein at different stringency levels in controlling for false positives. **c** The number of Bonferroni <0.05, positive significant microbiome associations by cell type. **d** The human genes, per cell type, with the greatest number of microbial associations that themselves had low or Bonferroni-significant *p* values (Two-sided). Each point in the plot bodies represent a different bacterial species (top) or viral genus (bottom). For each cell type, we ranked genes with non-zero LASSO coefficients first by the number of Bonferroni < 0.2 findings then by the total number of nominally associated (*p* value < 0.05) microbial features (bacteria or viruses). We report up to ten human genes per sub-panel. Source data are provided through the github link.

Given our observation that spaceflight-associated immune change bears a resemblance to infection, we next aimed to identify if there were observable correlations between DEGs and specific bacterial and viral microbiome features as a function of flight. Specifically, the abundance of bacterial and viral taxonomies correlated with DEGs was examined across all cell types. We used a regression-based approach (See Methods) to illuminate relationships between DEGs and bacterial/viral taxonomic abundances across 10 body sites derived from shotgun metatranscriptomic and metagenomic sequencing (Supplementary Data 13)^58^. We compared the results of our associations across regularized (LASSO) regression and linear mixed models across different normalization methods and different taxonomic classifiers and ranks, finding overall concordant results (Supplementary Figs. 14, 15). We note that this effort is meant to be exploratory; the overall low sample size and number of statistical tests being executed could inflate false positives. It is for this reason we emphasize (Fig. 5) total magnitude of FDR/nominally significant associations per cell type and opt not to focus heavily on specific host gene/microbiome gene interactions.

Numerous genes known for involvement in immune response to bacterial and viral invasion were highly correlated to microbiome abundance changes before and after flight. GO term overrepresentation analysis indicated enrichment (padj < 0.05) for diverse pathways related to bacterial and viral response and immune cell change (Fig. 5a, Supplementary Data 14). Null hypothesis testing on randomized data had limited overlap with the results from the real analysis, both in terms of LASSO coefficients as well as nominal and Bonferroni-adjusted p-values (Fig. 5b**)**. When considering only genes that were Bonferroni significant and had non-zero lasso coefficients, CD14 monocytes had the most bacterial associations (Fig. 5c). Viral associations were, by comparison, predominantly in NK cells and T cells. As such, the enrichment of pathogen-response pathways linked to microbial features indicates that microbiome shifts may drive a portion of immune response to spaceflight. Various genes for cell proliferation (i.e., *STAT1*), ribosomal genes, and various pathways of pathogen infection (*RPL28*, *RPS27A*, *RPL10*, *RPL13*, *RPL11*, *RPSA*, *UBA52*) were associated with bacteria (Fig. 5d, top panel), notably in CD14 monocytes. Viruses (Fig. 5d, bottom panel) were also associated with genes involved in inflammatory response but with some distinct associations. These included inflammatory cytokine signaling (*JAK2*, *STAT3*, *STAT1*), immune cell differentiation (*HLA-DQA2*, *HLA-DPA1*), and immune cell adhesion (*ITGA4*). Viruses were notably associated with *HLA* gene expression across CD16 monocytes, NK cells, and DCs.

## Discussion

This study provides the most comprehensive immune profiling of astronauts to date, including single-cell multi-omics data, antibody isotypes, cfRNA abundance, BCR/TCR diversity, TFBS enrichments, sex-dependent changes, microbiome interactions, integration of other astronaut and mouse missions, and potential countermeasures. The I4 crew showed several cytokine and gene regulatory changes that mirrored longer-duration missions, including increased IL-6, IL-10, and MCP-1 and post-flight decreases in MHC class I genes. The post-flight decrease in MHC class I genes was conserved in the I4 immune cells, I4 plasma cfRNA, JAXA plasma cfRNA, and NASA Twins Study immune cells. Of note, the ‘spaceflight signatures of I4 astronauts’ were also conserved in the I4 immune cells, the NASA Twins Study, and the meta-analysis of 27 GeneLab mouse spaceflight studies, which consistently showed dysregulated OXPHOS, translation, UV response, and TCF21 pathways (Supplementary Data 15). Moreover, these data showed a pronounced sex- and cell-type specificity regarding responsiveness and recovery from spaceflight, with the most and least sensitive cells being CD14 monocytes and CD16 monocytes and T cells, respectively. With these results, the I4 data enabled a unique molecular portrait of a civilian crew with a more diverse genetic and biomedical background than most NASA crews, which are generally restrictive cohorts.

While promising in terms of cellular recovery profiles, the I4 mission was not designed to determine the safety of spaceflight for all civilians, nor should these data alone be used to make judgments for future crew selection, number, age, sex, or fitness for spaceflight. Moreover, some functions of chromatin accessibility or gene regulation differences are inferred (e.g., chromVAR), and will need additional characterization in future studies. Nonetheless, the molecular measures from the I4 crew enabled new comparisons to prior missions (e.g., the NASA Twins Study, NASA JSC data, JAXA cfRNA) and meta-analysis of the space-flown mouse studies (817 samples across 27 datasets encompassing 10 different mouse tissues), which mostly confirmed our observations for the significantly altered pathways. Overall, a rapid gene and chromatin recovery was seen for the crew across most cell types, save for the CD14 monocytes and 16 monocytes, and these data can serve as additional baseline biomedical data and context for future missions. Repeated measures of pre- and post-flight metrics of crews will enable better contextualization and statistical rigor by researchers and clinicians and enable guidance for how astronauts can serve as their own controls (within-subjects design) by using their pre-flight immune marker levels as a reference.

A limitation of this study was the relatively low number of participants, which is a common challenge in spaceflight studies. While the collection workflows and timing were standardized to minimize variation (e.g., morning blood draws, crew training, and oversight during collection), some cell and sample variations can still occur. As such, our results focused on significantly shifted genes and pathways consistently observed across all crew members. In addition, we utilized a battery of other studies to validate our observations, including other astronaut data, NASA Twins Study data, JAXA CFE data, in vitro microgravity-simulated PBMCs, I4 cfRNA data, meta-analysis of the GeneLab mouse studies, as well as other omics data from the I4 mission. Of particular interest are the differential changes between I4 PBMCs and the in vitro-simulated microgravity PBMCs, which enabled a novel way to differentiate the likely impact of radiation vs. microgravity on human PBMCs, which indicates that radiation is a likely larger driver of the cellular responses. Of course, simulated cells can still be influenced by other experimental factors (e.g., space radiation, fluid shifts, environment, temperature), and future studies should seek to confirm and expand these DEGs related to radiation vs. microgravity.

Sex-specific variation in immune response is frequently observed in clinical settings, but poorly understood, and this phenomenon has yet to be investigated in-depth at the single-cell level during spaceflight. Here, we observed that males appear to be more affected by spaceflight, for almost all cell types and metrics, experiencing more DEGs, slower recovery to the baseline of DEGs, and a slower recovery of the chromatin accessibility. In addition, IL-6, IL-8, and fibrinogen differences by sex were confirmed in the replication cohort of NASA astronauts (*n* = 64). Sex differences also were observed in certain *CD* and *HLA* genes (Supplementary Data 9, 10), including a greater number of downregulated *HLA* genes in male DCs (Supplementary Data 10). For example, CD83 is expressed on mature dendritic cells, and it was downregulated in male DCs at *R* + 1 (Supplementary Data 10). Under simulated microgravity, DCs had decreased expressions of HLA-DR and impaired phagocytosis^59^, which aligns with our results. While dysregulation of immune function, oxidative phosphorylation, and Myc target pathways were conserved in both males and females, the aggregate data thus far indicates that the gene regulatory and immune response to spaceflight is more sensitive in males. More studies will be needed to confirm these trends, but such results can have implications for recovery times and possibly crew selection (e.g., more females) for high-altitude, lunar, and deep space missions.

We additionally observed increased expression in many immune cell pathways associated with pathogens. Immune cell activity is known to be specifically associated with shifts in host bacterial and viral content^60–63^. Microgravity has the potential to affect gene regulations in various tissues, including interactions between the host immune system and microbiome^64^. Bacteria can directly regulate immune cell activity and viral activation^65–68^, and herpes virus reactivation is associated with spaceflight^69^. When associating microbiome features with DEGs, we identified that many of the pathways most associated with bacterial and viral shifts before and after flight were additionally related to pathogen colonization. As a result, we hypothesize that a portion of the immune activation in flight could be arising from microbiome shifts. However, our microbiome integration results are descriptive and, in future studies, could be further strengthened by mechanistic experiments, targeted microbiome interventions, and larger sample sizes. Further, although we used techniques standard for the field, other microbiome analytic methods (i.e., gene-level analysis and other machine-learning approaches) could tease further signals from the data. Indeed, the substantial differences between bacterial and viral responses indicate discrete mechanisms of immune response and provide targets for future studies.

In conclusion, these convergent signatures of spaceflight begin to detail the core, consistent set of human cellular and molecular responses to spaceflight and can help narrow the targets for countermeasures and monitoring in future studies. Moreover, any potential long-term changes need to be monitored longitudinally to scan for any reversion or exacerbation of the phenotypes before pharmacological or biomedical interventions can be considered. Exploring the data introduced here is enabled through our interactive data portal (https://soma.weill.cornell.edu) and downloadable through public and controlled-access repositories (NASA Open Science Data Repositories and GeneLab/ALSDA). In the coming years, NASA plans to put the first woman and first person of color on the Moon, and such ambitious missions can leverage the data shown here, which details cell-, person-, and sex-specific molecular data, thus guiding more precise risk mitigation, crew health monitoring, and long-term countermeasure development.

## Methods

### IRB statement human subjects research

All subjects were consented at an informed consent briefing at SpaceX (Hawthorne, CA), and samples were collected and processed under the approval of the Institutional Review Board (IRB) at Weill Cornell Medicine, under Protocol 21-05023569. All crew members have consented to data and sample sharing. Tissue samples were provided by SpaceX Inspiration4 crew members after consent for research use of the biopsies, swabs, and biological materials. The procedure followed guidelines set by the Health Insurance Portability and Accountability Act and operated under IRB-approved protocols. Experiments were conducted in accordance with local regulations and with the approval of the IRB at Weill Cornell Medicine (IRB #21-05023569).

### PBMC isolation and single-cell library preparation

For each crew member, 8 mL of venous blood was collected in EDTA anticoagulant tubes. Depletion of granulocytes was performed either directly from whole blood using the RosetteSepTM granulocyte depletion cocktail or by cell sorting after PBMC isolation. Whole blood was incubated in a granulocyte depletion cocktail (50 µL/mL of blood) for 20 min at room temperature. Next, Ficoll-Paque Plus (Cytiva) was utilized to isolate PBMCs by density gradient centrifugation. After washes in PBS with 2% FBS (GIBCO) were completed, isolated PBMCs were cell sorted to remove granulocytes only if the RosetteSepTM granulocyte depletion cocktail was not added to whole blood prior to density gradient centrifugation. Granulocytes were identified using side scatter and the lymphocyte and monocyte fractions were sorted using a 100 µm nozzle (BD Aria). Following granulocyte depletion, PBMCs were split into two fractions to generate single cell V(D)J T-cell and B-cell libraries or multiomic (GEX and ATAC) libraries. To capture T-cell and B-cell V(D)J repertoire, single cell gel beads-in-emulsion and libraries were performed according to the manufacturer’s instructions (Chromium Next GEM Single Cell 5′ v2, 10X Genomics). Prior to single cell multiome ATAC and gene expression sequencing, nuclei isolation was performed by resuspending PBMCs in 100 µL of cold lysis buffer containing 10 mM Tris-HCl (pH 7.4), 10 mM NaCl, 3 mM MgCl_2_, 0.1% Tween-20, 0.1% Nonidet P40, 0.01% digitonin, 1% BSA, 1 mM DTT and 1 U/µL RNAse inhibitor. Cells were incubated for 4 min on ice, followed by the addition of 1 mL cold wash buffer (10 mM Tris-HCl (pH 7.4), 10 mM NaCl, 3 mM MgCl_2_, 0.1% Tween-20, 1% BSA, 0.1% Tween-20, 1 mM DTT, 1 U/µL RNAse inhibitor). After centrifugation (500 × *g* for 5 min at 4 °C), nuclei were resuspended in a diluted nuclei buffer (10X Genomics Single Cell Multiome ATAC kit A) at a concentration of 6000 nuclei per µL. Single-cell libraries were generated via the Chromium Next GEM Single Cell Multiome ATAC and Gene Expression kit (10X Genomics) according to the manufacturer’s instructions.

### Sequencing

In total, six timepoints were sequenced, including 92 days pre-flight (June), 44 days pre-flight (August), pre-flight (Pre-September), post-flight (Post-September), 45 days post-flight (November), 82 days post-flight (December). The libraries sequenced for each timepoint included ATAC, GEX, and VDJ (TCR + BCR) libraries. Four libraries corresponding to the four astronauts were prepared for each timepoint and library type, except for the June and August timepoints for which VDJ libraries were not prepared. In total, there were 24 ATAC and GEX libraries and 32 VDJ libraries, which were sequenced in three batches. The concentration of each library was taken using the Invitrogen Qubit 1X dsDNA HS Assay Kit and run on Agilent Tapestation 2100 using Agilent Technologies D1000 reagents and Screentape for fragment analysis. The nanomolarity of each sample was calculated using the formula ((concentration (ng/µl)/ (660 g/mol)* fragment size (bp))*10^6. After the nanomolarity was obtained for each sample, the target nanomolarity was determined using the lowest nanomolarity of the sample libraries. The desired number of reads per sample was determined based on the following criterion: 35,000 read pairs per cell for ATAC libraries, 25,000 read pairs per cell for GEX libraries, and 5000 read pairs per cell for VDJ libraries. Following this, the total number of reads per pool of samples was determined by adding all the read pairs per sample, and the percentage of total read pairs of each pool that is made up of each sample was determined using this formula (desired read pairs for sample/total read pairs for the pool) × 100. The target volume (µL) for each pool was determined based on requirements for sequencing, final molarity, etc. In this case, 200 µL was used for the ATAC library pool, and 100 µL was used for both the VDJ and GEX library pools. The target volume (µL) for each sample was then calculated based on the percentage of the total pool calculated earlier, using the desired final pool volume. Based on the target nanomolarity, sample nanomolarity, and sample target volume, the input volume (uL) for each sample was calculated using the following formula ((target nanomolarity * sample target volume)/sample nanomolarity). If the volume of each individual sample does not meet the target sample volume the rest of the volume can be made up using nuclease-free water. The total volume of both the samples and nuclease-free water should add up to the target pool volume. Following these pooling protocols, the samples were sequenced on the NovaSeq 6000 sequencing system.

### Alignment

GEX and ATAC libraries were processed using Cell Ranger arc v2.0.0. VDJ libraries were processed using Cell Ranger v6.1.1. Reads were aligned to the GRCh38 human genome. We generated single-cell combined transcriptome and ATAC data from PBMCs from individuals with Inspiration4 mission crews across three pre-flight (June 2021: L-92, August 2021: L-44, September Pre-launch: L-3) and three postflight (September Post-launch: *R* + 1, November 2021: *R* + 45, December 2021: *R* + 82). We mapped the single-cell multi-ome (GEX + ATAC) data using 10X chromium cellranger-arc (10X Genomics). We followed the 10X single-cell multi-ome analysis pipeline as previously reported and adpated for this data as decribes in this Methods section^11^. 151,411 cells passed quality control after quality control (minimum of 200 genes, maximum of 4500 gene counts, maximum of 20% mitochondrial reads, maximum of 100,000 peak counts, maximum of two nucleosome signal, and minimum of TSS enrichment per cell). Data were integrated using Harmony^70^.

### Processing of single-cell data

To identify putative cell types, Azimuth (version 0.3.2) pipeline was used with the reference dataset of Human. We annotated PBMCs subpopulations by supervised analysis guided by the Azimuth PBMC reference generated from single-cell transcriptome and CITE-seq^71^. For cellular proportion comparison after spaceflight, a p-value less than 0.05 with Wilcoxon rank sum test is considered significant. Differentially expressed genes and differentially accessible regions were identified with the FindMarkers function in Seurat (v4.2.0) packages (|log2FC | > 0.25 and padj < 0.05 with the default setting for other parameters). FindMotifs function in Seurat packages with default settings was used to identify the enriched transcription factor based on DARs. By running chromVAR, we computed a per-cell motif activity score (chromVAR deviation z-score of TF motifs) to visualize motif activities per cell. GeneActivity function in Signac package with default settings was used to quantify gene activity from ATAC, which generate a rough estimate of the transcriptional activity of each gene by quantifying ATAC-seq counts in the 2 kb-upstream region and gene body. AddModuleScore function in Seurat package with default setting was used to calculate the average expression levels of each program (cluster) on single cell level, subtracted by the aggregated expression of control feature sets. TCR and BCR-aligned data were analyzed with the VGenes package (https://github.com/WilsonImmunologyLab/VGenes).

### Biochemical profiling (BCP) and complete blood count (CBC)

Complete blood count (CBC) and comprehensive metabolic panels were completed by Quest Diagnostics. One 4 mL tube of whole blood collected in a K2 EDTA tube was used for the CBC, test code 6399. 500 µL of serum from a serum separator tube (SST) was submitted for the comprehensive metabolic panel (CMP), test code 10231. Serum samples were submitted to Eve Technologies for the biomarker profiling panels (1) Human Cytokine/Chemokine 71-Plex Discovery Assay® Array (HD71) and (2) Human Cardiovascular Disease Panel 3 9-Plex Discovery Assay® Array (HDCVD9). Concentration values were extrapolated using a four of five parameter logic standard curve. Samples were normalized by the mean of the preflight value of each crew. For BCP and CBC, a *p* value less than 0.05 with the Wilcoxon Rank Sum Test is considered significant. We analyzed the previously reported astronaut BCPs^57^ by further delineating the effect of sex- and time by Two-way ANOVA with a post hoc Bonferroni t-test.

### Fluorescence-activated cell sorting (FACS) of immune cells

The granulocyte-depleted peripheral blood samples were subjected to fluorescence-activated cell sorting to sort out different subsets of immune cells. Briefly, the frozen samples were thawed, and washed in cell staining buffer (Biolegend, cat no.420201). The cells were resuspended and incubated with monocyte blocking solution (Biolegend, cat no. 426102) and Fc Receptor Blocking Solution (Biolegend, cat no. 422301) for 20 min, followed by incubation with CD3 (T cells) (BD, cat no. 555342), CD19 (B cells) (Biolegend, cat no.302207), CD14 (Monocyte) (BD, cat no. 563420), and CD56 (for NK cells) (BD, cat no. 564058) for 30 min, washed twice in the cell staining buffer and resuspended in the same buffer. Right before sorting DAPI was added to eliminate dead cells during sorting. The gating strategy is shown in Supplementary Fig. 3d. The different immune cells were directly sorted into RNAlater for RNA extraction for further validation studies.

### Reverse transcription and quantitative PCR (qPCR)

FACS-isolated T cells were used for RNA extraction with RNeasy Mini kit (Qiagen, cat no.74134). cDNA was synthesized with SuperScript™ III First-Strand Synthesis System (Thermo Fisher Scientific, cat no. 18080051). TaqMan™ Fast Advanced system (Thermo Fisher Scientific, cat no. 4444557 used for qPCR quantification. qPCR primers are as follows.

• ACTB: Thermo Fisher Scientific, cat no.4331182, assay ID: Hs01060665_g1

• FOXP3: Thermo Fisher Scientific, cat no.4453320, assay ID: Hs01085834_m1

• TNFRSF9: Thermo Fisher Scientific, cat no.4453320, assay ID: Hs00155512_m1

• IL1R1: Thermo Fisher Scientific, cat no.4453320, assay ID: Hs00991010_m1

• IL1R2: Thermo Fisher Scientific, cat no.4453320, assay ID: Hs00174759_m1

• ENTPD1: Thermo Fisher Scientific, cat no.4453320, assay ID: Hs00969556_m1

• IL2RA: Thermo Fisher Scientific, cat no.4453320, assay ID: Hs00907777_m1

• VPS51: Thermo Fisher Scientific, cat no.4448892, assay ID: Hs00203146_m1

• VPS52: Thermo Fisher Scientific, cat no.4448892, assay ID: Hs00224000_m1

• VPS53: Thermo Fisher Scientific, cat no.4448892, assay ID: Hs00217606_m1

• VPS54: Thermo Fisher Scientific, cat no.4448892, assay ID: Hs00212957_m1

### Meta-analysis of GeneLab RNA-seq data from 817 samples across 27 datasets encompassing ten different mouse tissues

FASTQ files were programmatically downloaded from GeneLab (https://genelab.nasa.gov/) and processed using the MTD pipeline^72^. A meta-analysis of the GeneLab data was performed in two steps. Firstly, we performed batch correction using Combat-seq^73^ to standardize data across different datasets. Subsequently, DESeq2^74^ was used to perform differential expression analysis between the spaceflight and ground control mice while adjusting for age, sex, tissue, sacrifice site, and mission duration. The meta-analysis was done using DeSeq2 (wald test, two-tailed) based on batch(study)-corrected count matrix calculated with combat-seq. Multiple testing adjustment was done using Benjamini-hochberg. This analysis resulted in a ‘spaceflight signature in mice’ consisting of 2184 differentially expressed genes (1288 up-regulated and 896 down-regulated). To visually confirm that the genes in the spaceflight signature were changing in a similar direction across datasets based on differential expression analysis on each dataset separately grouping by tissue, sex, age, and mission duration.

### Principal component analysis

Prcomp function of R was used to calculate principal components from gene expression and peak expression profiles.

### Enrichment analysis

#### ssGSEA

ssGSEA package was used to calculate the normalized enrichment score with MSigDB pathways. For T cell activity score,

GO:2000566,GO:0043372,GO:0043378,GO:0043382,GO:0042104,GO:0045585,GO:0045588,GO:2000516,GO:2000568,GO:1903905,GO:2000563,GO:1900281,GO:2001187,GO:2001193,GO:0033091,GO:0045591,GO:2000451,GO:2000454,GO:1905404 were used. For B cells activity score, GO:0050871,GO:2000538,GO:0002663,GO:0002904,GO:0030890,GO:0045579,GO:0050861 were used. For NK cell activity score, GO:2000503,GO:0043323,GO:0032816,GO:0032819,GO:0032825,GO:0045954,GO:0002717,GO:0002729,GO:0002857,GO:0002860 were used. For monocytes activity score, GO:0042117,GO:0045657,GO:2000439,GO:1900625,GO:0090026,GO:0071639,GO:0030887 were used. For dendritic cell scores, GO:0030887,GO:2001200,GO:2000670,GO:2000549,GO:2000529,GO:2000510,GO:0002732,GO:0002735,GO:0002606 were used.

#### fgsea

fGSEA package was used to calculate the normalized enrichment score and the adjusted p-value with MSigDB Hallmark, MSigDB C2, MSigDB C5, and custom pathways generated with ranked gene sets from various datasets. Here is an example of the pre-ranked GSEA pipeline we performed. (a). Prepare the pathway collection In our case, we used MSigDB, “spaceflight signature of mice”, or DEGs from the selected study (b). Prepare the ranked list of genes. We ranked the gene sets by the adjusted *p* value, a combination of log2FC multiplied by −log10 (adjusted *p* value (or *p* value)), or stat. There are several ways to rank the gene sets by correlation, *p* value, adjusted *p* value, log2FC, stat, or combination of log2FC and adjusted *p* value (or *p* value) derived from the differential expression analysis. (c). Run GSEA

#### Over-representation analysis

gProfiler (https://biit.cs.ut.ee/gprofiler/) was used to identify significantly enriched (padj < 0.05) GO or KEGG pathways. All DEGs, up-regulated DEGs, downregulated DEGs, microbiome-associated DEGs were used as input.

### Canonical pathway analysis

Data were analyzed through the use of Ingenuity Pathway Analysis (IPA, version 01-22-01) (QIAGEN Inc.) software with DEGs, FDR < 0.05. Common DEGs between females and males and sex-specific DEGs were used for canonical pathways.

### Gene and pathway overlap analysis

#### I4 and GeneLab mouse data comparison

To compare the spaceflight signature in mice against the gene expression changes in Inspiration 4, we mapped the mouse genes to their human equivalent using Ensembl Biomart^75^, which resulted in 1942 human orthologues (1147 upregulated and 795 downregulated). These human orthologues were then compared against differentially expressed (padj < 0.05) in I4.

#### Fisher’s exact test

To calculate the significance of the overlap we used Fisher’s exact test implemented in the R package GeneOverlap (https://github.com/shenlab-sinai/GeneOverlap). *P* values for the overlaps were adjusted by multiple tests using Benjamini-Hochberg method^76^. We found that 80% of the comparisons in level 1 cell annotation were statistically significant, while 56% were significant in level 2 annotation. Notably, all comparisons involving down-regulated genes in the spaceflight signature in mice and I4 showed statistical significance. None of the comparisons between down-regulated gene sets in Inspiration 4 against up-regulated genes in the spaceflight signature in mice (and vice versa) yielded statistical significance in either level 1 or 2 cell annotations.

#### GSEA-based overlap analysis

By GSEA, ranking the genes from the GeneLab meta-analysis by the negative logarithm of the p-value multiplied by the sign of the fold change (i.e., 1 if positive, −1 if negative) and evaluating whether genes differentially expressed in each cell type in I4 were enriched in either direction of the ranked ordered list. For this calculation, we used the R package fgsea^77^.

#### I4 skin spatial transcriptomics

Skin biopsy samples from the four crew members were collected and frozen in cryovials pre and post flight. Collected skin was flash embedded in OCT blocks. Four replicate of OCT-embedded tissues were placed on a single slide, per astronaut,including pre and post flight replicates. Tissues were then cryosectioned at 5 µm thickness and attached to glass microscope slides (Fisher Scientific, cat# 22-037-246). Immunofluorescent visualization marker for Pan-Cytokeratin (PanCK, Novus cat# NBP2-33200, Alexa Fluor® 532, clone ID AE1 + AE3), fibroblast activation protein (FAP, Abcam cat# ab222924, Alexa Fluor® 594, clone ID EPR20021) and smooth muscle actin (SMA, R&D Systems cat# IC1420R, Alexa Fluor® 647, clone 1A4) were used for region or interest (ROI) selection. The DSP whole transcriptome assay (WTA) was used to assess genes collected in each ROI. For DSP processing, OCT slides were thawed overnight in 10% neutral-buffered formalin (NBF) at 4 °C followed by PBS washes for thorough fixation. After washes, slides were prepared following the automated Leica Bond RNA Slide Preparation Protocol for fixed frozen samples, digesting samples with 1.0 µg/mL proteinase K for 15 min, and antigen retrieval for 20 min at 100 °C (NanoString, no. MAN-10115-05). In situ hybridizations with the GeoMx Whole Transcriptome Atlas Panel (WTA, 18,677 genes) at 4 nM final concentration were done in Buffer R (NanoString). Morphology markers were prepared for four slides concurrently using Syto13 (DNA), PanCK, FAP and SMA in Buffer W for a total volume of 225 μl per slide. Slides incubated with 225 µl of morphology marker solution at RT for 1 h, then washed in SSC and loaded onto the NanoString DSP instrument. The 20× scan was used to select freeform ROIs to guide selection of outer epidermal (OE), inner epidermal (IE), outer dermal (OD) and vascular (VA) regions. OE ROIs covered spinous and granular layers, while IE ROIs covered the basal layer, identified from the staining of the tissue. To ensure proper selection and to avoid overlaps across different ROI types, small gaps between each ROI type were made. GeoMx WTA sequencing reads from NovaSeq6000 were compiled into FASTQ files corresponding to each ROI. FASTQ files were then converted to digital count conversion files using the NanoString GeoMx NGS DnD Pipeline. Additional methods can be found in ref. ^42^.

#### Metagenomics and metatranscriptomics DNA/RNA isolation, sequencing, and analysis

Briefly, we ran all metagenomic and metatranscriptomic samples through standard microbiome quality control pipelines using bbtools^78^ (deduplication, quality trimming, adapter removal, human read decontamination), and then computed taxonomic abundances with a variety of methods, those relevant to this manuscript being Xtree aligning to the Genome Taxonomy Database r207^79^, Xtree aligning to the complete set of non-redundant genomes in GenBank, MetaPhlAn4^80^, and Phanta^81^. We report results from all four of these approaches in the manuscript at the phylum, genus, and species ranks.

We used two approaches to identify associations between differentially expressed human genes and bacteria/viruses. First, we used Lasso regression to identify relationships between all DEGs for a given cell type and microbial features, characterizing any non-zero Lasso coefficient as a “potential association.” We use this phrasing because Lasso regression does not implicitly include statistical inference, therefore not controlling for false positive rates. We fit models of the form:

1 $$\,{humangene} \sim {microb}{e}_{1}+...+{microb}{e}_{n}$$

We fit different models for bacteria and viruses (e.g., for one model, microbe_1..x_ would be all bacteria, for another it would be all viruses). Prior to regressing, we centered and scaled the human gene abundances. We computed lasso models with both log transformed microbial abundances as well as Center-Log-Ratio transformed abundances, adding a pseudocount in the form of the smallest abundance value for a given matrix beforehand in both cases.

As a second form of association identification with the added benefit of statistical inference to control for false positives, we used a mixed effect linear regression approach for each microbe/DEG pair that had a non-zero coefficient coming out of the Lasso regressions. We transformed the gene/microbe abundances with the same approaches as above and fit models of the form:

2 $$\,{humangene} \sim {microbe}+(1{{{{{\rm{|}}}}}}{CrewID})$$

CrewID is a random effect corresponding to the different individuals meant to account for interindividual variation in microbial feature abundance. We used Bonferroni correction to adjust for multiple hypothesis testing, computing the threshold cutoff per cell type based on as the total number of microbes in a given rank and domain (e.g., all bacterial phyla, all bacterial genera, all bacterial species, all viral phyla, all viral genera) times the number of DEGs for a given cell type. This was meant to minimize the overall Bonferroni threshold. Additional methods for generating the bacterial and viral taxonomic abundance data used in the immune-microbiome associations are described in companion manuscripts^58,82^.

### I4 cfRNA processing

Plasma was frozen at −80 °C until processed. Before RNA extraction, plasma samples were thawed at room temperature and subsequently centrifuged at 1300 × *g* for 10 min at 4 °C. cfRNA was isolated from the plasma supernatant (300–800 µL) using the Norgen Plasma/Serum Circulating and Exosomal RNA Purification Mini Kit (Catalog No. 51000, Norgen). Next, 10 mL of DNase Turbo Buffer (Catalog No. AM2238, Invitrogen), 3 mL of DNase Turbo (Catalog No. AM2238, Invitrogen), and 1 mL of Baseline Zero DNase (Catalog No. DB0715K, Lucigen-Epicenter) was added to the extracted RNA and incubated for 30 min at 37 °C. Subsequently, the treated RNA was concentrated into a final volume of 12 mL with the Zymo RNA Clean and Concentrate Kit (Catalog No. R1015, Zymo).

Sequencing libraries prepared from 8 μL of concentrated RNA using the Takara SMARTer Stranded Total RNA-Seq Kit v3—Pico Input Mammalian (634485, Takara) and barcoded using the SMARTer RNA Unique Dual Index Kit (634451, Takara). Library concentration was quantified using a Qubit 3.0 Fluorometer (Q33216, Invitrogen) with the dsDNA HS Assay Kit (Q32854, Invitrogen). Libraries were quality-controlled using an Agilent Fragment Analyzer 5200 (M5310AA, Agilent) with the HS NGS Fragment kit (DNF-474-0500, Agilent). Libraries were pooled to equal concentrations and sequenced at Cornell Genomics on an Illumina NextSeq 2000 machine using 150-base pair, paired-end sequencing for an average of 26 million reads per sample.

Sequencing data was processed using a custom bioinformatics pipeline utilizing the Snakemake workflow management system (v7.7.0). Reads were quality filtered and trimmed using BBDUK (v38.90), aligned to the Gencode GRCh38 human reference genome (v38, primary assembly) using STAR (v2.7.0f) default parameters, deduplicated using UMI tools (v1.1.2), and features quantified using featureCount (v2.0.0). Mitochondrial, ribosomal, X, and Y chromosome genes were removed prior to analysis. cfRNA sample quality was determined by calculating DNA contamination (intron/exon ratio), rRNA contamination, number of feature counts, and RNA degradation. All samples passed QC. Read counts of technical duplicate samples were combined for downstream analyses.

Cell-type deconvolution was performed using BayesPrism (v2.0) with the Tabula Sapiens single cell RNA-seq atlas (Release 1)^83,84^. Cells from the Tabula Sapiens atlas were grouped as previously described in ref. ^85^. Comparative analysis of DEGs was performed using a negative binomial model as implemented in the DESeq2 package (v1.34.0) using a Benjamini-Hochberg corrected *p* value cutoff < 0.05, unless otherwise stated. Variance stabilization transformation was used for comparing and plotting gene counts, unless otherwise stated.

### I4 secretome data

Plasma samples were centrifuged at 12,000 × *g* for 20 min and then Extracellular Vesicles and Particles (EVPs) were collected by ultracentrifugation at 100,000 × *g* for 70 min. EVPs were then washed in PBS and again collected by ultracentrifugation at 100,000 × *g* for 70 min. The final EVP pellet was resuspended in PBS. Two micrograms of enriched EVPs were digested and analyzed with LC-MS/MS in data-dependent acquisition mode. The list of differentially abundant proteins from the plasma proteomics data was filtered for differentially expressed genes that had an adjusted *p* value < 0.05 and |logFC | > 1. Heatmaps were generated with mitochondrial and oxidative stress genes using R package pheatmap. Additional protocol details on EVP proteomic profiling is described in ref. ^86^.

### NASA twins study RNA-seq data

Gene set enrichment analysis and gene expression data have been downloaded from the previous publication^7^. In brief, one astronaut was monitored before, during, and after a 1-year mission onboard the ISS, and his identical twin sibling was also monitored at the same time, serving as a genetically matched ground control for this study.

### JAXA CFE cfRNA data

Plasma cell-free RNA differential expression data and study protocols were shared through NASA’s GeneLab platform with accession number GLDS-530/OSD-530. Briefly, blood samples were collected from 6 astronauts before, during, and after the spaceflight on the ISS. Total RNA was purified from plasma samples and processed for RNA-seq analysis. Mean expression values were obtained from normalized read counts of 6 astronauts for each time point.

### Single-cell microgravity simulated in vitro PBMCs

Single-cell RNA-seq data was generated from donor PBMCs exposed to simulated microgravity (μG) for 25 h, with a continual motion machine (Rotating Wall Vessel) serving as an in vitro model for microgravity. DEGs were calculated as μG vs. 1 G (control) for pooled data after two donor sequencing reactions (one male and one female). Detailed methods can be found in ref. ^44^.

### Bulk RNA-sequencing from mouse tissues

All mouse RNA-sequencing datasets used were previously published and are publicly available. Brown adipose tissue, kidney, liver, mandibular bone, spleen, temporal bone, thymus, white adipose tissue and soleus were collected from mice that had spent 31 days on the ISS, two days after returning to Earth^87,88^. A different group of soleus samples were collected from mice that had spent 34.1 days in microgravity, 3.3 days after returning to Earth^89^. The tibialis anterior samples were obtained from the Rodent Research-23 mission. Briefly, mice were maintained in microgravity for 38 days and were euthanized and dissected after 24 hours from returning to Earth. A more thorough description of the experimental design can be found at NASA GeneLab, Doi: 10.26030/zw7z-bj40. Raw reads were trimmed galore, then aligned using STAR2 alignment to mm10 or Salmon to Ensembl transcripts. Gene counties were obtained using featurecounts. All RNAseq differential expression analyses were performed using DESeq2(1.38.3) in RStudio (R, 4.2.3). Heatmaps and volcano plots were produced using the R packages ComplexHeatmap (2.15.1) and EnhancedVolcano (1.16.0), respectively.

### Compound analysis

FDA-approved drugs (*n* = 1692) are selected from the DrugBank database and food compounds (*n* = 7962) are selected from the FoodDB database. LINCS compounds (*n* = 5414) are obtained from LINCS L1000 project. “Compound” is used as a general term for “drug”, “food compound” and “LINCS compound” throughout the document.

Compound-protein interactions are extracted from the STITCH database v5.069 by matching the InChI keys of drugs/food/LINCS compounds. STITCH collects information from multiple sources and individual scores from each source are combined into an overall confidence score. After processing, three data sets are obtained: i) drug-gene interaction dataset containing 1890 drugs and 16,654 genes with 542,577 interactions ii) food compound - gene interaction dataset containing 7,654 compounds and 116,375 genes and 818,737 interactions iii) LINCS compound—gene interaction dataset containing 5414 compounds and 16,794 genes and 692,152 interactions. The universal gene set contains all genes that interact with at least one compound. The compound with low p-value interacts with a higher proportion of the DEGs than that expected by chance. Statistically significant compounds were then obtained after Bonferroni adjustment of *p* values. The pipeline for this compound analysis is implemented in the gcea R package (https://github.com/nguyenkhiemv/gcea).

### Further protocol and sample processing information

Sample collection methods and data generation have been detailed in full in the paper^86^.

### Reporting summary

Further information on research design is available in the Nature Portfolio Reporting Summary linked to this article.

### Supplementary information

Supplementary Information

Peer Review File

Description of Additional Supplementary Files

Supplementary Data 1-15

Reporting Summary

### Source data

Source Data

## Data Availability

Datasets generated in this study have been deposited in the NASA Open Science Data Repositories (OSDR; osdr.nasa.gov; comprised of GeneLab^90^ and the Ames Life Sciences Data Archive [ALSDA]^2,91^). Identifiers for publicly downloadable datasets in the OSDR are documented below. Single-cell data can be visualized online through the SOMA Data Explorer: https://epigenetics.weill.cornell.edu/apps/I4_Multiome/. Source data are provided with this paper (PBMC: OSD-570, Blood Serum: OSD-575). Source data are provided with this paper.

## Code availability

All code used to generate Figures and analyses from this project is available at https://github.com/eliah-o/inspiration4-omics.
