# Supplementary material for: Single-cell multi-ome and immune profiles of the Inspiration4 crew reveal conserved, cell-type, and sex-specific responses to spaceflight
Source: Nat Commun. 2024 Jun 11;15:4954. doi: 10.1038/s41467-024-49211-2 (PMC11166952; doi:10.1038/s41467-024-49211-2)
Supplement: Supplementary file 1 — Supplementary Information [file 41467_2024_49211_MOESM1_ESM.pdf]

Supplementary Figures

Supplementary Figure 1

a, Pie chart of significantly increased, significantly decreased, and stable BCPs. b, The percentage change of the significantly increased BCPs (Top) and decreased BCPs (Bottom) over time. Each dot represents the value of each crew (Wilcoxon-rank sum test, p-value < 0.05, two-sided). c, Percent change of the selected biochemical profiles in the <3 day (i4 mission), < 6 months (28 astronauts), and 1 year (NASA Twins study). d, Expression profiles of the comprehensive metabolic blood panel (CMP) over time. Each dot represents the value of each crew. e, Dot plot of 18 significantly changed BCPs (from Supplementary Fig. 1b) in pseudo-bulk PBMC. Source data are provided as a Source Data file.

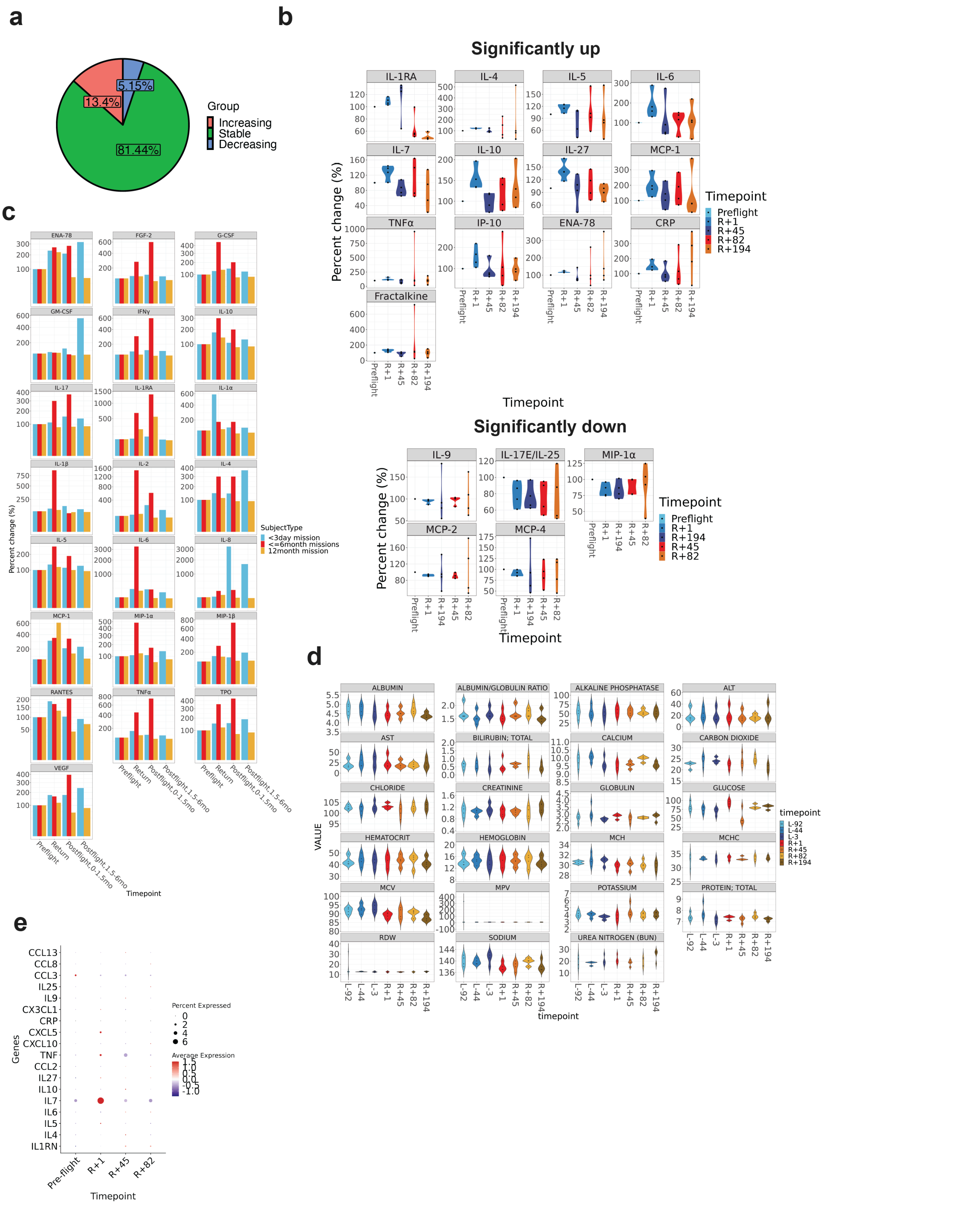
a
b
Significantly up
Significantly down
c
d
e
Group
Increasing
Stable
Decreasing
13.4%
5.15%
81.44%
IL-1RA
IL-4
IL-5
IL-6
IL-7
IL-10
IL-27
MCP-1
TNFα
IP-10
ENA-78
CRP
Fractalkine
IL-9
IL-17E/IL-25
MIP-1α
MCP-2
MCP-4
Percent change (%)
Timepoint
Preflight
R+1
R+45
R+82
R+194
SubjectType
<3day mission
<=6month missions
12month mission
Return
Postflight.0-1.5mo
Postflight.1.5-6mo
ALBUMIN
ALBUMIN/GLOBULIN RATIO
ALKALINE PHOSPHATASE
ALT
AST
BILIRUBIN; TOTAL
CALCIUM
CARBON DIOXIDE
CHLORIDE
CREATININE
GLOBULIN
GLUCOSE
HEMATOCRIT
HEMOGLOBIN
MCH
MCHC
MCV
MPV
POTASSIUM
PROTEIN; TOTAL
RDW
SODIUM
UREA NITROGEN (BUN)
VALUE
timepoint
L-92
L-44
L-3
Percent Expressed
Average Expression
Genes
Pre-flight
CCL13
CCL8
CCL3
IL25
IL9
CX3CL1
CRP
CXCL5
CXCL10
TNF
CCL2
IL27
IL10
IL7
IL6
IL5
IL4
IL1RN

Supplementary Figure 2

a, Heatmap of the transcriptomic expression in mouse tissues of the cytokines found significantly changed in the serum of the i4 crew. b-d Volcano plots of the mouse tissues in which the cytokines shown in panel (a) were found differentially expressed after landing. e-f, Heatmaps with the expression of the cytokines in panel (a) in the soleus muscle comparing flight (FLT) to ground control (GC) in the datasets PMID 34168270 (e) and PMID:33911096 (f). g-h, Volcano plots comparing gene expression between flight and ground control in the soleus from PMID 34168270 (g) and PMID:33911096 (h). i. Heatmap of the expression in the tibialis anterior muscle (OSD-576) of the cytokines found significantly changed in the serum of the i4 crew. Wald test (two-sided) was used to identify differentially expressed genes, with raw p-values, (*) means p-value <0.05 in panels e,f, and i. Source data are provided as a Source Data file.

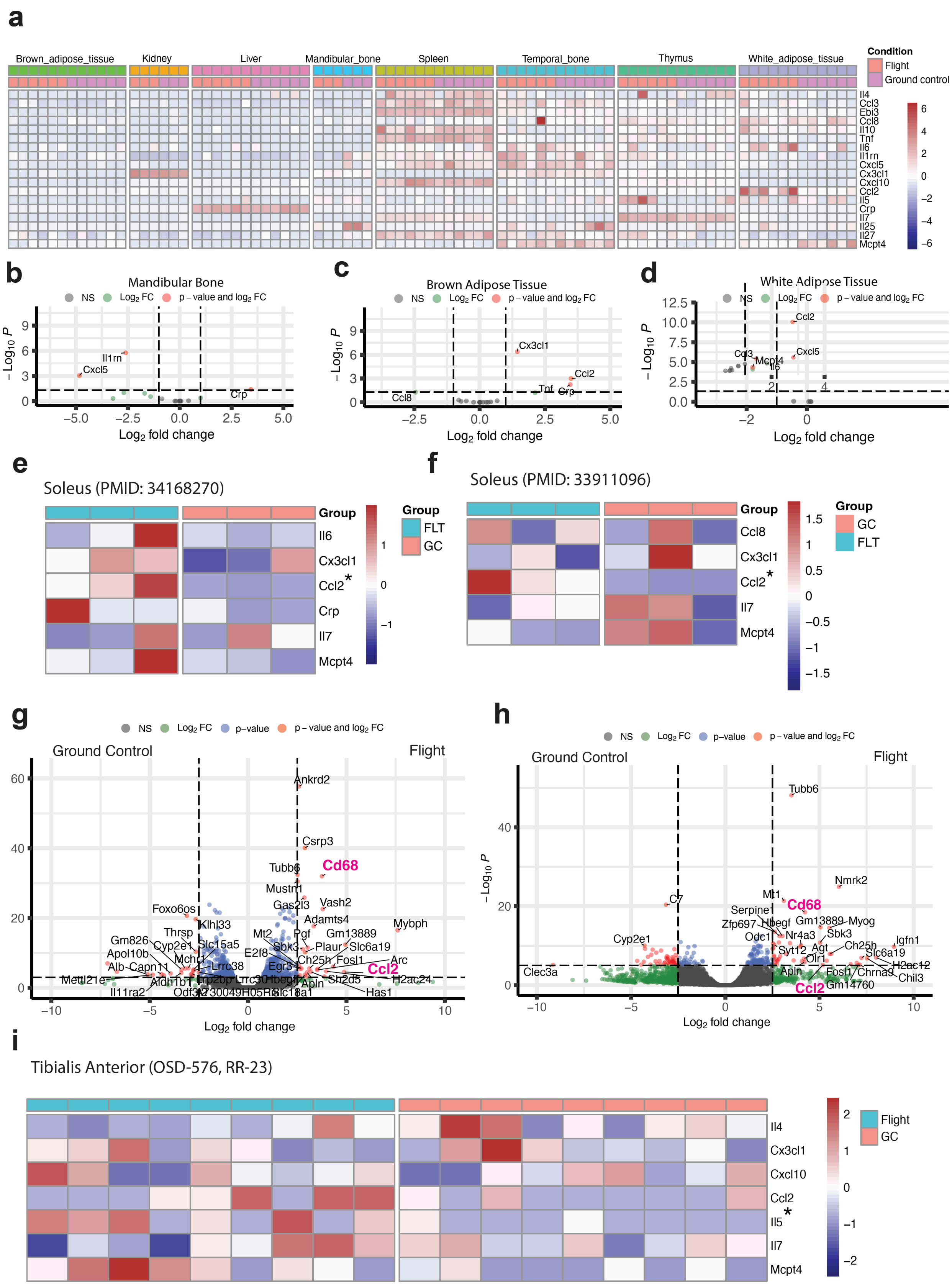
a
Brown_adipose_tissue
Kidney
Liver
Mandibular_bone
Spleen
Temporal_bone
Thymus
White_adipose_tissue
Condition
Flight
Ground control
Il4
Ccl3
Ebi3
Ccl8
Il10
Tnf
Il6
Il1rn
Cxcl5
Cx3cl1
Cxcl10
Ccl2
Il5
Crp
Il7
Il25
Il27
Mcpt4
b
Mandibular Bone
NS
Log2 FC
p − value and log2 FC
− Log10 P
Il1rn
Cxcl5
Crp
Log2 fold change
c
Brown Adipose Tissue
Cx3cl1
Ccl2
Tnf
Crp
Ccl8
d
White Adipose Tissue
Ccl2
Ccl3
Mcpt4
Il6
Cxcl5
e
Soleus (PMID: 34168270)
Group
FLT
GC
Il6
Cx3cl1
Ccl2*
Crp
Il7
Mcpt4
f
Soleus (PMID: 33911096)
Group
GC
FLT
Ccl8
Cx3cl1
Ccl2*
Il7
Mcpt4
g
NS
Log2 FC
p−value
p − value and log2 FC
Ground Control
Flight
Ankrd2
Csrp3
Tubb6
Cd68
Mustn1
Gas2l3
Vash2
Foxo6os
Klhl33
Adamts4
Mybph
Thrsp
Mt2
Pgf
Gm13889
Gm826
Cyp2e1
Slc15a5
Sbk3
Plaur
Slc6a19
Apol10b
E2f8
Mchr1
Lrrc38
Ch25h
Arc
Alb
Capn11
Egr3
Fosl1
Ccl2
Mettl21e
Aldh1b1
Has1
H2ac24
Il11ra2
Odf3l2
Sh2d5
Apln
Slc18a1
Log2 fold change
h
NS
Log2 FC
p−value
p − value and log2 FC
Ground Control
Flight
Tubb6
Nmrk2
C7
Mt1
Cd68
Serpine1
Zfp697
Hbegf
Gm13889
Myog
Cyp2e1
Odc1
Nr4a3
Sbk3
Igfn1
Syt12
Agt
Ch25h
Slc6a19
Olr1
H2ac12
Clec3a
Apln
Fosl1
Chrna9
Chil3
Ccl2
Gm14760
− Log10 P
Log2 fold change
i
Tibialis Anterior (OSD-576, RR-23)
Flight
GC
Il4
Cx3cl1
Cxcl10
Ccl2
Il5*
Il7
Mcpt4

Supplementary Figure 3

a, Activity score of PBMC subpopulation markers in the annotated i4 PBMC**.** b, UMAP of 151,411 cells derived from single-nuclei multi-ome (GEX+ATAC) with subpopulation annotation. c, Cellular profiles of i4 PBMC over time calculated from single-nuclei multi-ome data. Each dot represents each crew. d, FACS gating of T cell, B cell, NK cell, and monocytes from the i4 PBMC. e, Cellular profiles of i4 PBMC over time calculated from FACS. Paired Student's t-test. f, CBC profiles of i4 crews over time. Each dot represents each crew. Source data are provided as a Source Data file.

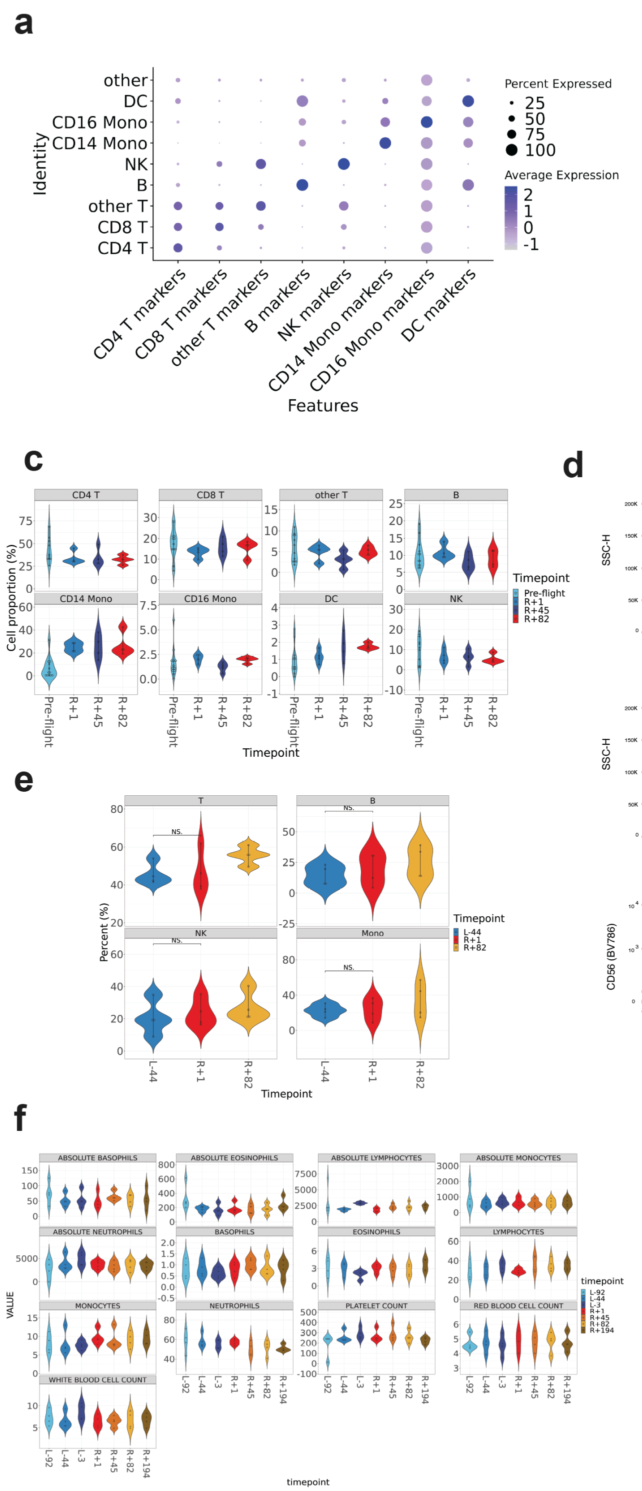
a
b
151,411 cells
c
d
e
f
Features
Identity
Percent Expressed
Average Expression
UMAP_1
UMAP_2
Cell proportion (%)
Timepoint
Pre
Landing
Post
Non-T
T-cells
Non-CD14 Cells
NK Cells
B-cells
CD3 (APC)
CD14 (BV650)
CD19 (PE)
CD56 (BV786)
SSC-H
Percent (%)
VALUE
timepoint

32

33

34

Supplementary Figure 4

Principal component analysis of single-nuclei GEX of the i4 immune cells. Shape represents ID. Color represents timepoints. Source data are provided as a Source Data file.

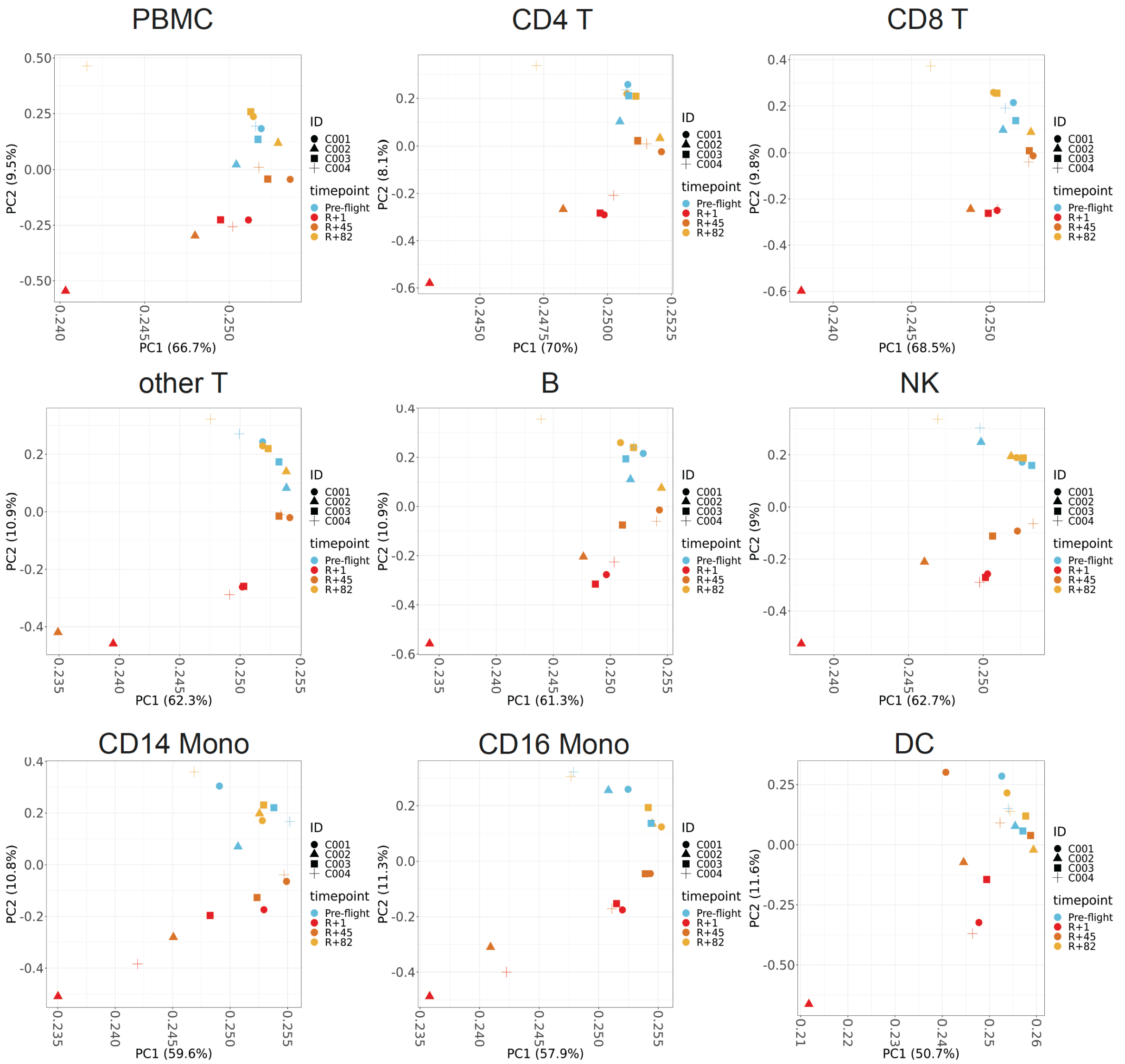

Supplementary Figure 5

Principal component analysis of single-nuclei ATAC of the i4 immune cells. Shape represents ID. Color represents timepoints. Source data are provided as a Source Data file.

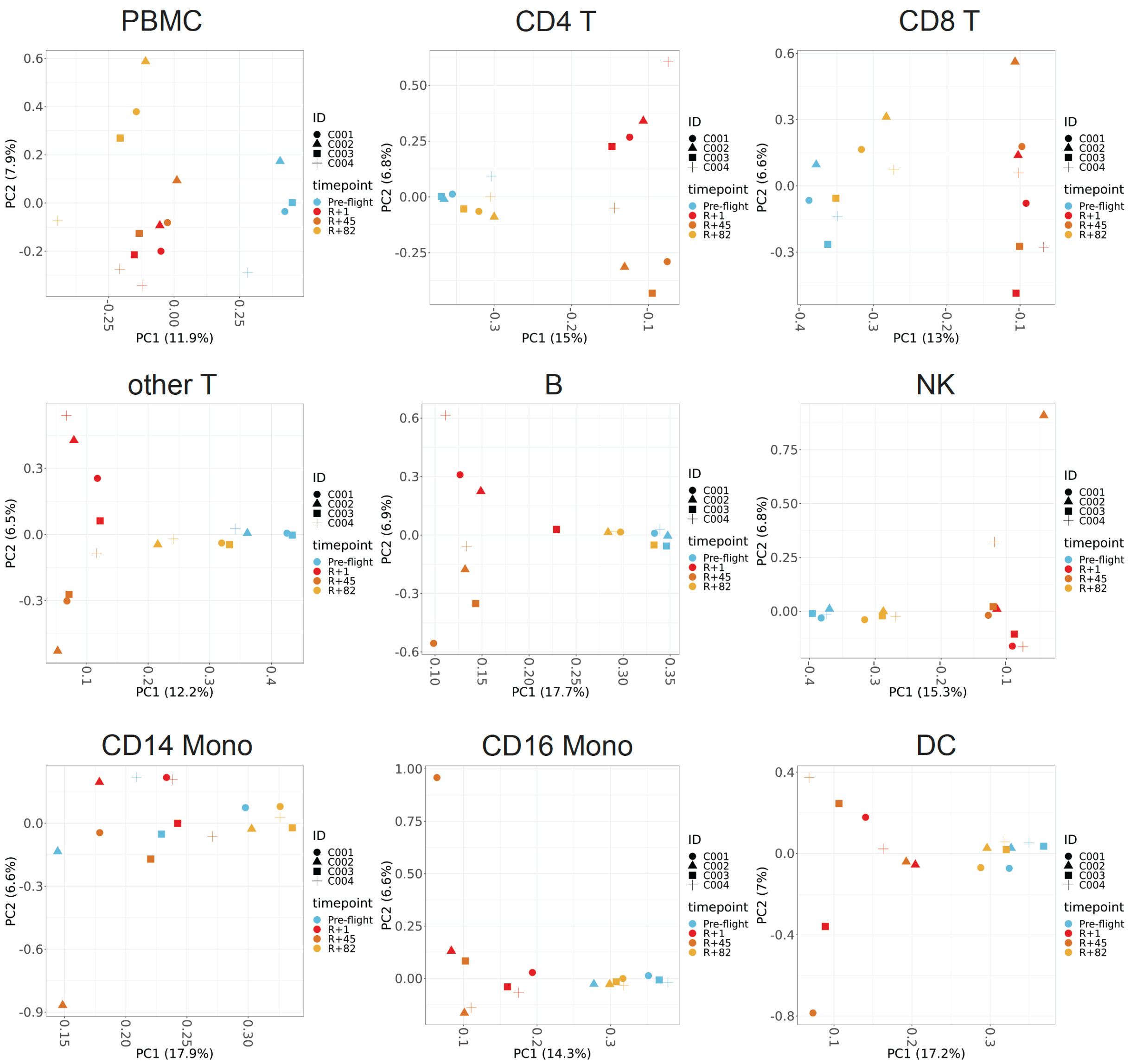

Supplementary Figure 6

a, Volcano plot of the pseudo-bulk PBMCs DEGs (Left) and DARs (Right). A Wilcoxon rank-sum test to identify differentially expressed genes between clusters, with raw p-values adjusted for multiple testing using the Bonferroni correction to control the family-wise error rate (FWER). b, The number of DEGs and DARs identified post-flight (R+1) and the long-term post-flights (R+45 and R+82) from PBMC and subpopulations. c, Overlap of up- and down-regulated DEGs and DARs among PBMC and subpopulations. d, GSEA analysis the i4 immune cells DEGs with the 'spaceflight signatures in the i4 astronauts' DEGs. A one-sided permutation-based test to determine the significance of gene set enrichment, with raw p-values adjusted for multiple testing using the Benjamini-Hochberg procedure to control the false discovery rate (FDR). e, Top 12 enriched DNA motifs of PBMC that are over-represented in a set of peaks that are differentially accessible between R+1 and pre-flight. Source data are provided as a Source Data file.

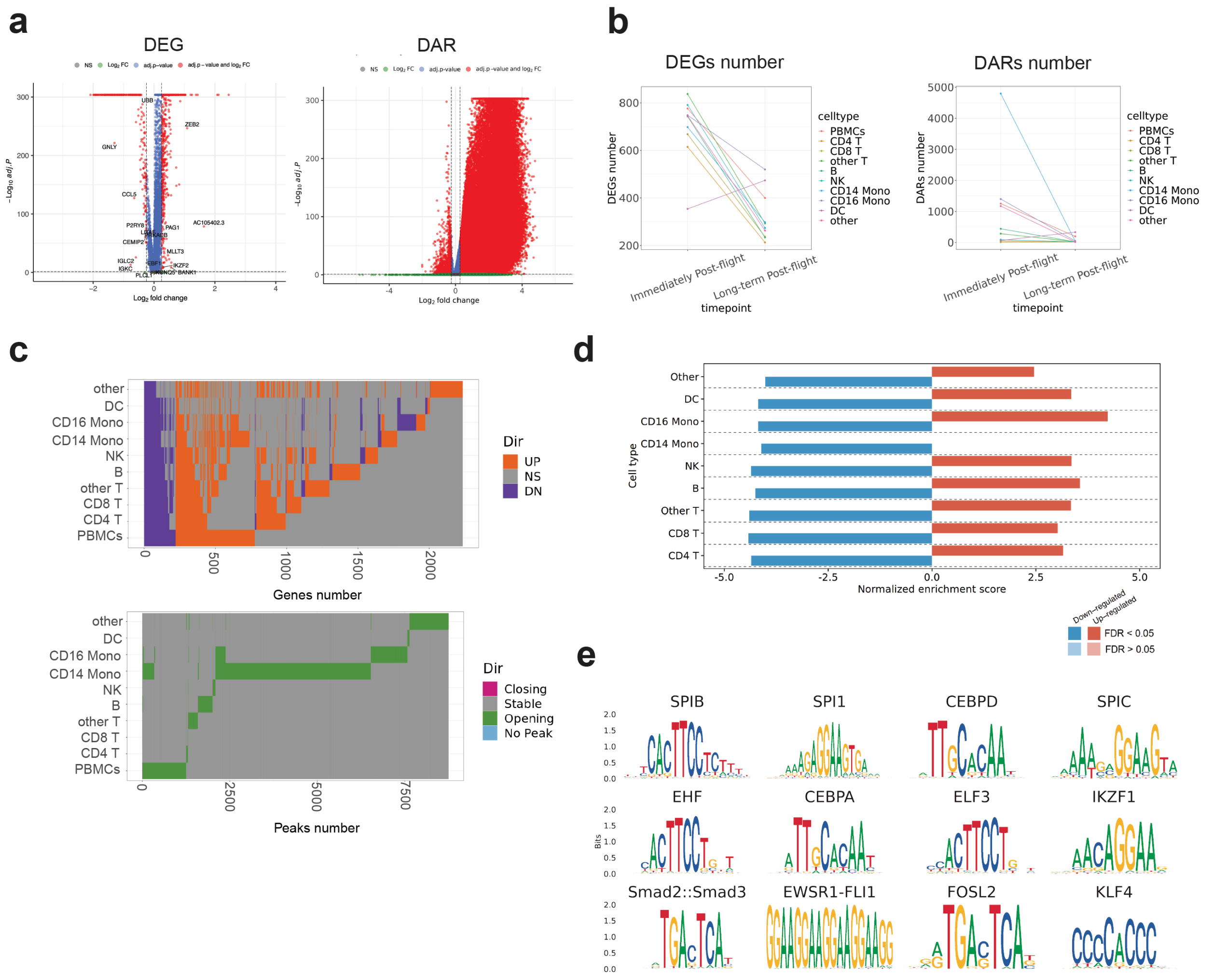

Supplementary Figure 7

a, Cellular profiles of T cells from single-nuclei multi-ome data over time. Each dot represents each crew. b, Cellular profiles of B cells from single-nuclei multi-ome data over time. Each dot represents each crew. c, Cellular profiles of NK cells from single-nuclei multi-ome data over time. Each dot represents each crew. d, Cellular profiles of i4 monocytes and DCs from single-nuclei multi-ome data over time. Each dot represents each crew. e, The number of total mutations in TCR from L-3 to R+82 (Wilcoxon-rank sum test, p-value < 0.05). f, The percentage of gene types of TCR from L-3 to R+82. g, The number of total mutations in BCR from L-3 to R+82 (Wilcoxon-rank sum test, *p-value < 0.05, ** p-value < 0.01). h, The percentage of antibody isotypes of BCR from L-3 to R+82. Source data are provided as a Source Data file.

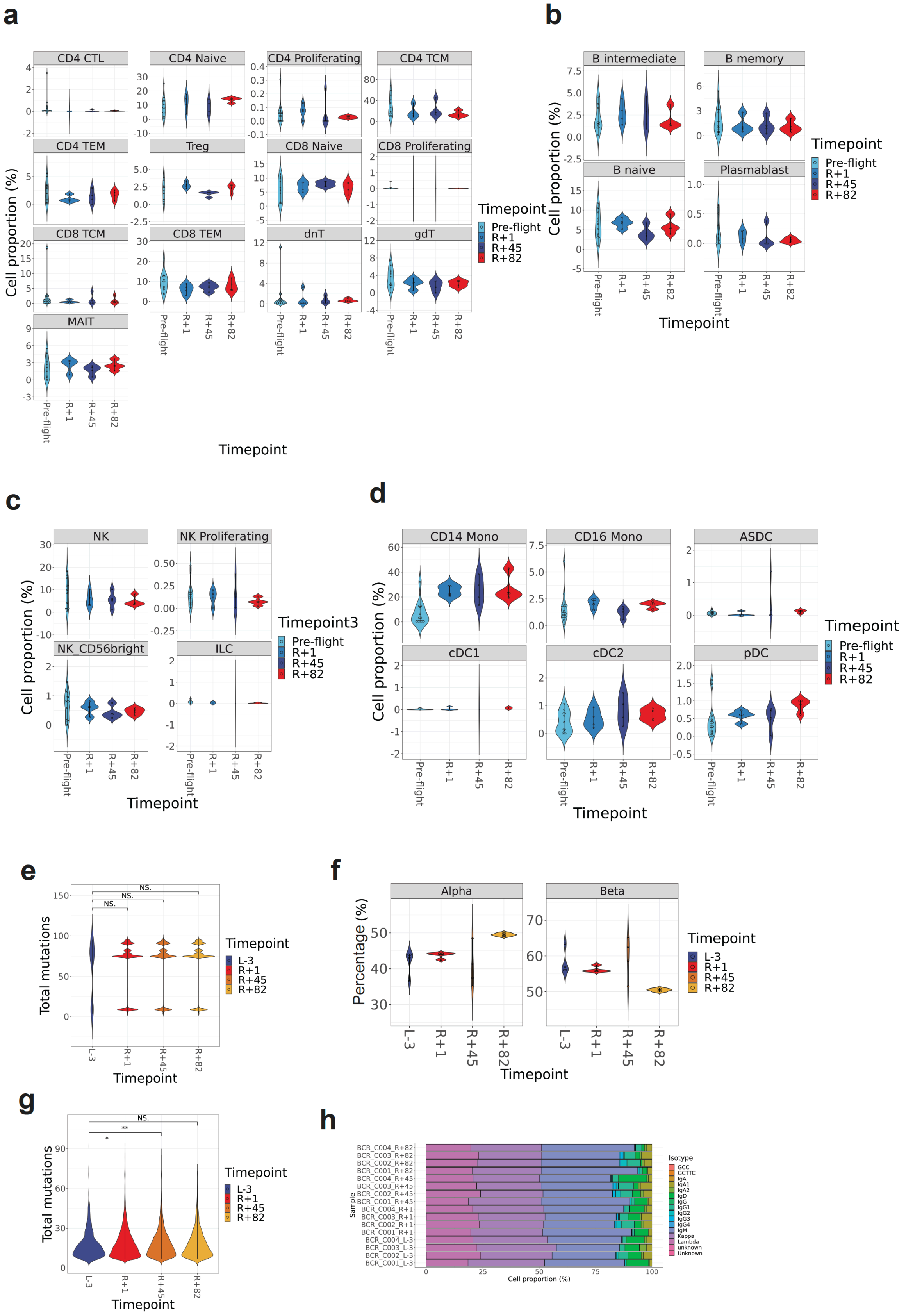
a
b
c
d
e
f
g
h
CD4 CTL
CD4 Naive
CD4 Proliferating
CD4 TCM
CD4 TEM
Treg
CD8 Naive
CD8 Proliferating
CD8 TCM
CD8 TEM
dnT
gdT
MAIT
B intermediate
B memory
B naive
Plasmablast
NK
NK Proliferating
NK_CD56bright
ILC
CD14 Mono
CD16 Mono
ASDC
cDC1
cDC2
pDC
Alpha
Beta
Cell proportion (%)
Total mutations
Percentage (%)
Timepoint
Timepoint3
Pre-flight
R+1
R+45
R+82
L-3
NS.
*
**
Sample
Cell proportion (%)
Isotype
GCC
GCTTC
IgA
IgA1
IgA2
IgD
IgG
IgG1
IgG2
IgG3
IgG4
IgM
Kappa
Lambda
unknown
Unknown
BCR_C004_R+82
BCR_C003_R+82
BCR_C002_R+82
BCR_C001_R+82
BCR_C004_R+45
BCR_C003_R+45
BCR_C002_R+45
BCR_C001_R+45
BCR_C004_R+1
BCR_C003_R+1
BCR_C002_R+1
BCR_C001_R+1
BCR_C004_L-3
BCR_C003_L-3
BCR_C002_L-3
BCR_C001_L-3

Supplementary Figure 8

Enrichment score of the relevant immune function pathways of T cell (top row), B cell (2nd row), NK cell (3rd row), monocytes (4th row), and dendritic cell (bottom row) subpopulations before (Pre-flight: L-92, L-44, L-3) and after spaceflight (Post-flight: R+1, R+45, R+82). Source data are provided as a Source Data file.

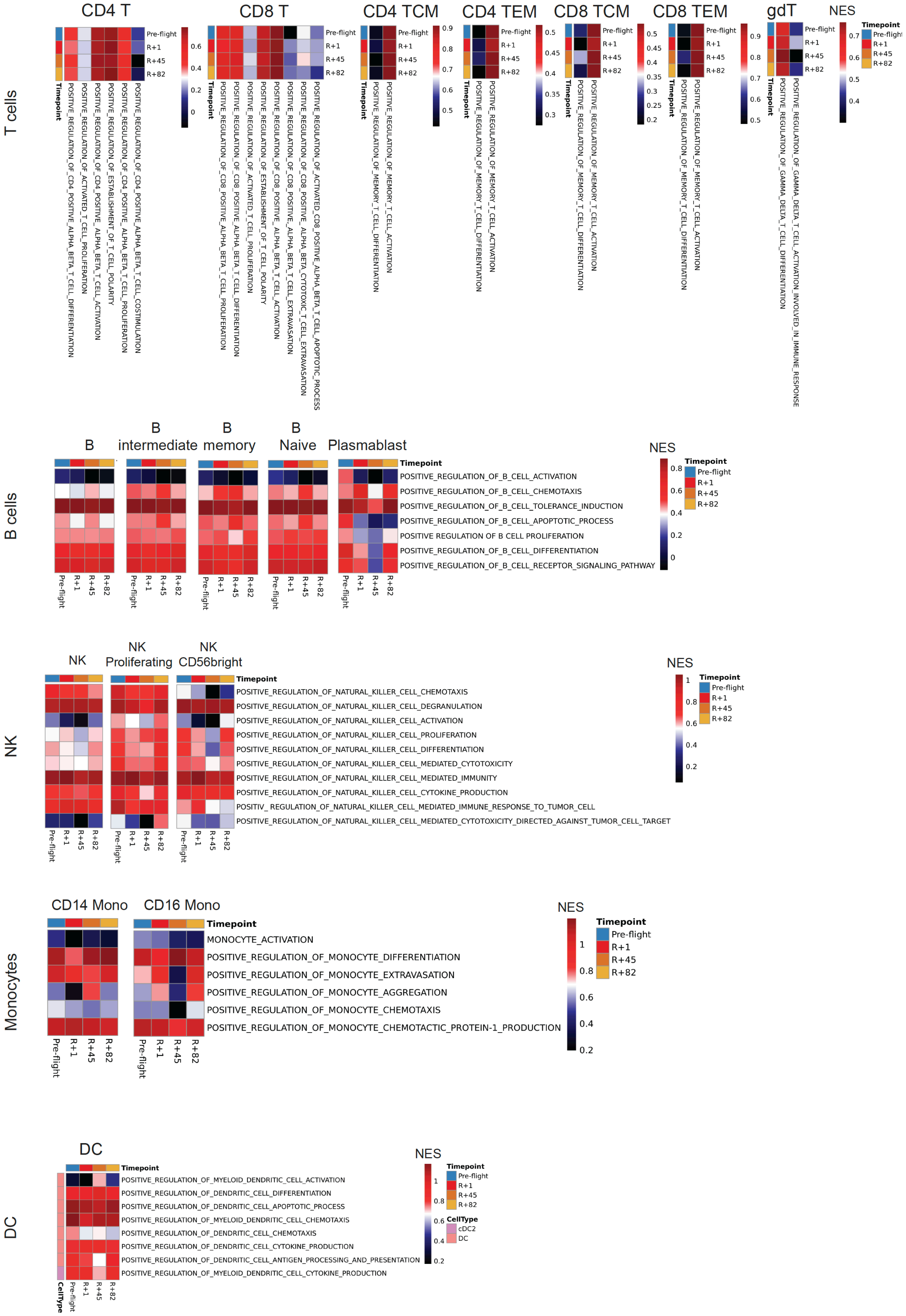
T cells
CD4 T
CD8 T
CD4 TCM
CD4 TEM
CD8 TCM
CD8 TEM
gdT
NES
Timepoint
Pre-flight
R+1
R+45
R+82
POSITIVE_REGULATION_OF_CD4_POSITIVE_ALPHA_BETA_T_CELL_DIFFERENTIATION
POSITIVE_REGULATION_OF_ACTIVATED_T_CELL_PROLIFERATION
POSITIVE_REGULATION_OF_CD4_POSITIVE_ALPHA_BETA_T_CELL_ACTIVATION
POSITIVE_REGULATION_OF_ESTABLISHMENT_OF_T_CELL_POLARITY
POSITIVE_REGULATION_OF_CD4_POSITIVE_ALPHA_BETA_T_CELL_PROLIFERATION
POSITIVE_REGULATION_OF_CD4_POSITIVE_ALPHA_BETA_T_CELL_COSTIMULATION
POSITIVE_REGULATION_OF_CD8_POSITIVE_ALPHA_BETA_T_CELL_PROLIFERATION
POSITIVE_REGULATION_OF_CD8_POSITIVE_ALPHA_BETA_T_CELL_DIFFERENTIATION
POSITIVE_REGULATION_OF_ACTIVATED_T_CELL_PROLIFERATION
POSITIVE_REGULATION_OF_ESTABLISHMENT_OF_T_CELL_POLARITY
POSITIVE_REGULATION_OF_CD8_POSITIVE_ALPHA_BETA_T_CELL_ACTIVATION
POSITIVE_REGULATION_OF_CD8_POSITIVE_ALPHA_BETA_T_CELL_EXTRAVASATION
POSITIVE_REGULATION_OF_CD8_POSITIVE_ALPHA_BETA_CYTOTOXIC_T_CELL_EXTRAVASATION
POSITIVE_REGULATION_OF_ACTIVATED_CD8_POSITIVE_ALPHA_BETA_T_CELL_APOPTOTIC_PROCESS
POSITIVE_REGULATION_OF_MEMORY_T_CELL_DIFFERENTIATION
POSITIVE_REGULATION_OF_MEMORY_T_CELL_ACTIVATION
POSITIVE_REGULATION_OF_GAMMA_DELTA_T_CELL_DIFFERENTIATION
POSITIVE_REGULATION_OF_GAMMA_DELTA_T_CELL_ACTIVATION_INVOLVED_IN_IMMUNE_RESPONSE
B cells
B
B intermediate
B memory
B Naive
Plasmablast
POSITIVE_REGULATION_OF_B_CELL_ACTIVATION
POSITIVE_REGULATION_OF_B_CELL_CHEMOTAXIS
POSITIVE_REGULATION_OF_B_CELL_TOLERANCE_INDUCTION
POSITIVE_REGULATION_OF_B_CELL_APOPTOTIC_PROCESS
POSITIVE REGULATION OF B CELL PROLIFERATION
POSITIVE_REGULATION_OF_B_CELL_DIFFERENTIATION
POSITIVE_REGULATION_OF_B_CELL_RECEPTOR_SIGNALING_PATHWAY
NK
NK Proliferating
NK CD56bright
POSITIVE_REGULATION_OF_NATURAL_KILLER_CELL_CHEMOTAXIS
POSITIVE_REGULATION_OF_NATURAL_KILLER_CELL_DEGRANULATION
POSITIVE_REGULATION_OF_NATURAL_KILLER_CELL_ACTIVATION
POSITIVE_REGULATION_OF_NATURAL_KILLER_CELL_PROLIFERATION
POSITIVE_REGULATION_OF_NATURAL_KILLER_CELL_DIFFERENTIATION
POSITIVE_REGULATION_OF_NATURAL_KILLER_CELL_MEDIATED_CYTOTOXICITY
POSITIVE_REGULATION_OF_NATURAL_KILLER_CELL_MEDIATED_IMMUNITY
POSITIVE_REGULATION_OF_NATURAL_KILLER_CELL_CYTOKINE_PRODUCTION
POSITIV_ REGULATION_OF_NATURAL_KILLER_CELL_MEDIATED_IMMUNE_RESPONSE_TO_TUMOR_CELL
POSITIVE_REGULATION_OF_NATURAL_KILLER_CELL_MEDIATED_CYTOTOXICITY_DIRECTED_AGAINST_TUMOR_CELL_TARGET
Monocytes
CD14 Mono
CD16 Mono
MONOCYTE_ACTIVATION
POSITIVE_REGULATION_OF_MONOCYTE_DIFFERENTIATION
POSITIVE_REGULATION_OF_MONOCYTE_EXTRAVASATION
POSITIVE_REGULATION_OF_MONOCYTE_AGGREGATION
POSITIVE_REGULATION_OF_MONOCYTE_CHEMOTAXIS
POSITIVE_REGULATION_OF_MONOCYTE_CHEMOTACTIC_PROTEIN-1_PRODUCTION
DC
POSITIVE_REGULATION_OF_MYELOID_DENDRITIC_CELL_ACTIVATION
POSITIVE_REGULATION_OF_DENDRITIC_CELL_DIFFERENTIATION
POSITIVE_REGULATION_OF_DENDRITIC_CELL_APOPTOTIC_PROCESS
POSITIVE_REGULATION_OF_MYELOID_DENDRITIC_CELL_CHEMOTAXIS
POSITIVE_REGULATION_OF_DENDRITIC_CELL_CHEMOTAXIS
POSITIVE_REGULATION_OF_DENDRITIC_CELL_CYTOKINE_PRODUCTION
POSITIVE_REGULATION_OF_DENDRITIC_CELL_ANTIGEN_PROCESSING_AND_PRESENTATION
POSITIVE_REGULATION_OF_MYELOID_DENDRITIC_CELL_CYTOKINE_PRODUCTION
CellType
cDC2
DC

Supplementary Figure 9

a, Dot plots of Th1-, Th2-, and Th17-secreted cytokines in CD4 TCM cells over time (Left: gene expression, Right: ATAC derived gene expression). b, GSEA of CD4 TCM cells with the MSigDB C7 GSE1924 for Th1, Th2, Th17 CD4 T cell gene expression signatures. A one-sided permutation-based test to determine the significance of gene set enrichment, with raw p-values adjusted for multiple testing using the Benjamini-Hochberg procedure to control the false discovery rate (FDR). c, GSEA of PBMC and subpopulations with MSigDB Hallmark pathway (padj < 0.05). A one-sided permutation-based test to determine the significance of gene set enrichment, with raw p-values adjusted for multiple testing using the Benjamini-Hochberg procedure to control the false discovery rate (FDR). d, Activity scores of top enriched motifs from PBMC subpopulations. Source data are provided as a Source Data file.

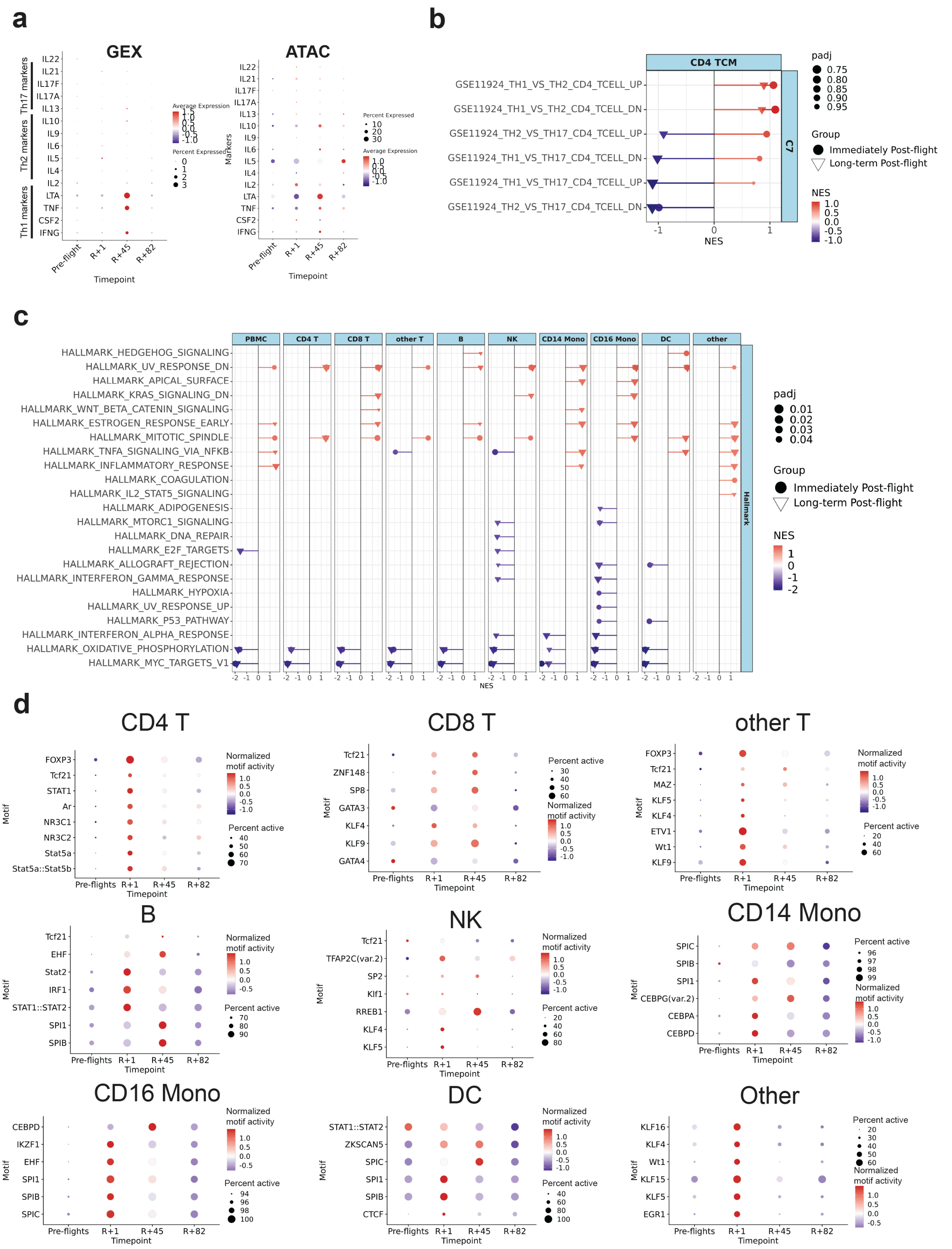

a
GEX
ATAC
Th17 markers
Th2 markers
Th1 markers
IL22
IL21
IL17F
IL17A
IL13
IL10
IL9
IL6
IL5
IL4
IL2
LTA
TNF
CSF2
IFNG
Average Expression
Percent Expressed
Markers
Pre-flight
R+1
R+45
R+82
Timepoint
b
CD4 TCM
GSE11924_TH1_VS_TH2_CD4_TCELL_UP
GSE11924_TH1_VS_TH2_CD4_TCELL_DN
GSE11924_TH2_VS_TH17_CD4_TCELL_UP
GSE11924_TH1_VS_TH17_CD4_TCELL_DN
GSE11924_TH1_VS_TH17_CD4_TCELL_UP
GSE11924_TH2_VS_TH17_CD4_TCELL_DN
C7
NES
padj
Group
Immediately Post-flight
Long-term Post-flight
c
PBMC
CD4 T
CD8 T
other T
B
NK
CD14 Mono
CD16 Mono
DC
other
Hallmark
HALLMARK_HEDGEHOG_SIGNALING
HALLMARK_UV_RESPONSE_DN
HALLMARK_APICAL_SURFACE
HALLMARK_KRAS_SIGNALING_DN
HALLMARK_WNT_BETA_CATENIN_SIGNALING
HALLMARK_ESTROGEN_RESPONSE_EARLY
HALLMARK_MITOTIC_SPINDLE
HALLMARK_TNFA_SIGNALING_VIA_NFKB
HALLMARK_INFLAMMATORY_RESPONSE
HALLMARK_COAGULATION
HALLMARK_IL2_STAT5_SIGNALING
HALLMARK_ADIPOGENESIS
HALLMARK_MTORC1_SIGNALING
HALLMARK_DNA_REPAIR
HALLMARK_E2F_TARGETS
HALLMARK_ALLOGRAFT_REJECTION
HALLMARK_INTERFERON_GAMMA_RESPONSE
HALLMARK_HYPOXIA
HALLMARK_UV_RESPONSE_UP
HALLMARK_P53_PATHWAY
HALLMARK_INTERFERON_ALPHA_RESPONSE
HALLMARK_OXIDATIVE_PHOSPHORYLATION
HALLMARK_MYC_TARGETS_V1
NES
padj
Group
Immediately Post-flight
Long-term Post-flight
d
CD4 T
CD8 T
other T
B
NK
CD14 Mono
CD16 Mono
DC
Other
Motif
Normalized motif activity
Percent active
Pre-flights
R+1
R+45
R+82
Timepoint

Supplementary Figure 10

a, Fisher's exact test of 'spaceflight signature in mice' with 'spaceflight signature in Inspiration4 astronauts'. P-values for the overlaps were adjusted by multiple tests using Benjamini-Hochberg method. b, Overlap percentage of GSEA of the 'spaceflight signatures in mice' with i4. The number on the bar represents the number of overlapping pathways (Fisher's exact test. padj; Hallmark: 3.844985e-02, C2: 3.317221e-47, C5: 2.341219e-83). P-values for the overlaps were adjusted by multiple tests using Benjamini-Hochberg method. c, GSEA of i4 PBMCs and subpopulations at the immediately post-flight (R+1) and long-term post-flights (R+45 and R+82) with up-regulated and down-regulated DEGs of NASA Twin study (padj < 0.05). LD: lymphocyte depleted. A one-sided permutation-based test to determine the significance of gene set enrichment, with raw p-values adjusted for multiple testing using the Benjamini-Hochberg procedure to control the false discovery rate (FDR). d, Overlap percentage of GSEA of the i4 spatial transcriptomics with the i4 immune cells. The number on the bar represents the number of overlapping pathways. Fisher's exact test. P-values for the overlaps were adjusted by multiple tests using Benjamini-Hochberg method. e, Overlap percentage of GSEA of the i4 EVP and plasma proteomics with the i4 immune cells. The number on the bar represents the number of overlapping pathways. Fisher's exact test. P-values for the overlaps were adjusted by multiple tests using Benjamini-Hochberg method. f, Altuna plot represents the overlap of up-regulated DEGs (Orange) and down-regulated DEGs (Purple) from i4 PBMCs and subpopulations and the core 375 DEGs of *in vitro* microgravity simulated PBMCs. g, Gene set enrichment analysis of 375 core DEGs of *in vitro* microgravity simulated DEGs with the MSigDB hallmark (Top, padj < 0.3). A one-sided permutation-based test to determine the significance of gene set enrichment, with raw p-values adjusted for multiple testing using the Benjamini-Hochberg procedure to control the false discovery rate (FDR). Source data are provided as a Source Data file.

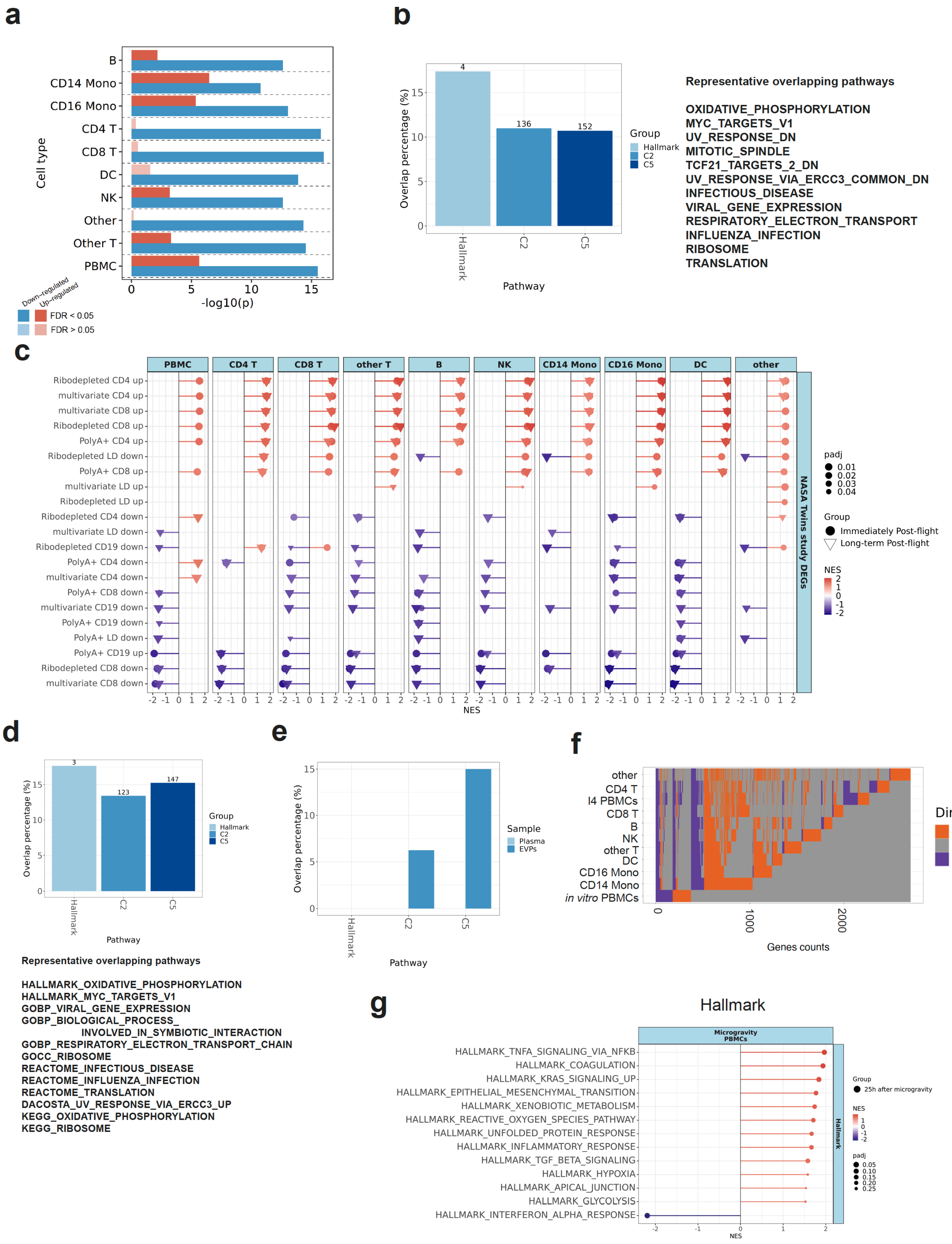
a
b
c
d
e
f
g
Cell type
-log10(p)
FDR < 0.05
FDR > 0.05
Down–regulated
Up–regulated
Overlap percentage (%)
Pathway
Group
Hallmark
C2
C5
Representative overlapping pathways
OXIDATIVE_PHOSPHORYLATION
MYC_TARGETS_V1
UV_RESPONSE_DN
MITOTIC_SPINDLE
TCF21_TARGETS_2_DN
UV_RESPONSE_VIA_ERCC3_COMMON_DN
INFECTIOUS_DISEASE
VIRAL_GENE_EXPRESSION
RESPIRATORY_ELECTRON_TRANSPORT
INFLUENZA_INFECTION
RIBOSOME
TRANSLATION
NASA Twins study DEGs
NES
padj
Immediately Post-flight
Long-term Post-flight
Representative overlapping pathways
HALLMARK_OXIDATIVE_PHOSPHORYLATION
HALLMARK_MYC_TARGETS_V1
GOBP_VIRAL_GENE_EXPRESSION
GOBP_BIOLOGICAL_PROCESS_
INVOLVED_IN_SYMBIOTIC_INTERACTION
GOBP_RESPIRATORY_ELECTRON_TRANSPORT_CHAIN
GOCC_RIBOSOME
REACTOME_INFECTIOUS_DISEASE
REACTOME_INFLUENZA_INFECTION
REACTOME_TRANSLATION
DACOSTA_UV_RESPONSE_VIA_ERCC3_UP
KEGG_OXIDATIVE_PHOSPHORYLATION
KEGG_RIBOSOME
Sample
Plasma
EVPs
Dir
UP
NS
DN
Genes counts
Hallmark
Microgravity PBMCs
25h after microgravity

Supplementary Figure 11

a, MHC class I gene expression in JAXA CFE plasma cfRNA. Error bar represents the SEM. b, log2FoldChange and q-value of MHC class I genes in CD4 T and CD8 T cells of NASA Twins study. Negative binomial test with adjustment only within each comparison separately. c, MHC class I gene expression in Inspiration4 plasma cfRNA. Source data are provided as a Source Data file.

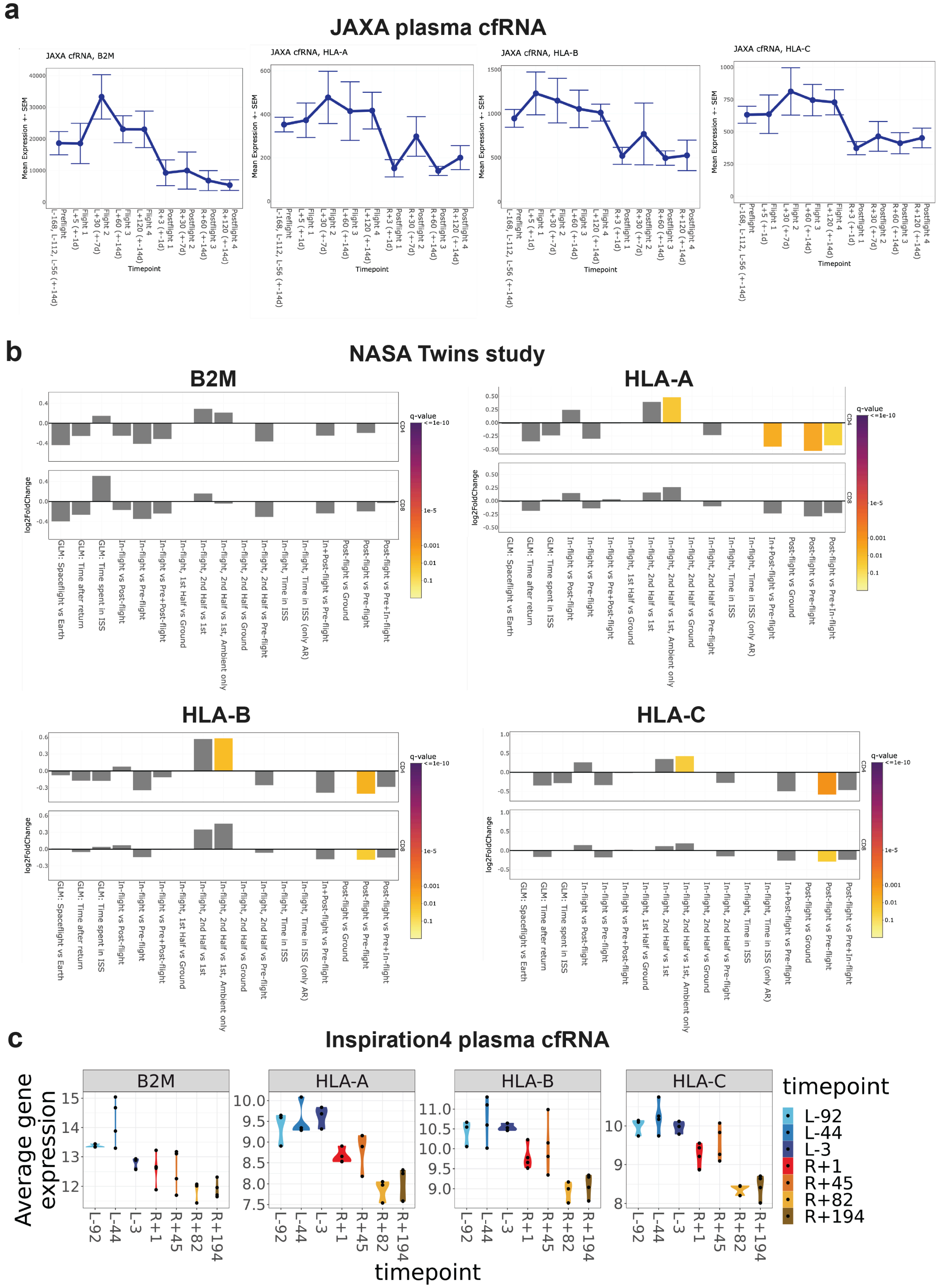
a
JAXA plasma cfRNA
JAXA cfRNA, B2M
JAXA cfRNA, HLA-A
JAXA cfRNA, HLA-B
JAXA cfRNA, HLA-C
Mean Expression +- SEM
Timepoint
b
NASA Twins study
B2M
HLA-A
HLA-B
HLA-C
log2FoldChange
q-value
c
Inspiration4 plasma cfRNA
B2M
HLA-A
HLA-B
HLA-C
Average gene expression
timepoint
L-92
L-44
L-3
R+1
R+45
R+82
R+194

Supplementary Figure 12

a, Dot plots of BCR signaling pathway scores in B cells over time. b, GSEA of B cells with the MSigDB hallmark and C2 pathway (C2: top7 of NES > 0 and NES <0, padj < 0.05). A one-sided permutation-based test to determine the significance of gene set enrichment, with raw p-values adjusted for multiple testing using the Benjamini-Hochberg procedure to control the false discovery rate (FDR). c, GSEA of T cells with the MSigDB hallmark and C2 pathway (C2: top7 of NES > 0 and NES <0, padj < 0.05). A one-sided permutation-based test to determine the significance of gene set enrichment, with raw p-values adjusted for multiple testing using the Benjamini-Hochberg procedure to control the false discovery rate (FDR). d, Dot plot of Treg markers and Treg activation markers in T cells (Left: gene expression. Right: ATAC derived gene expression). Source data are provided as a Source Data file.

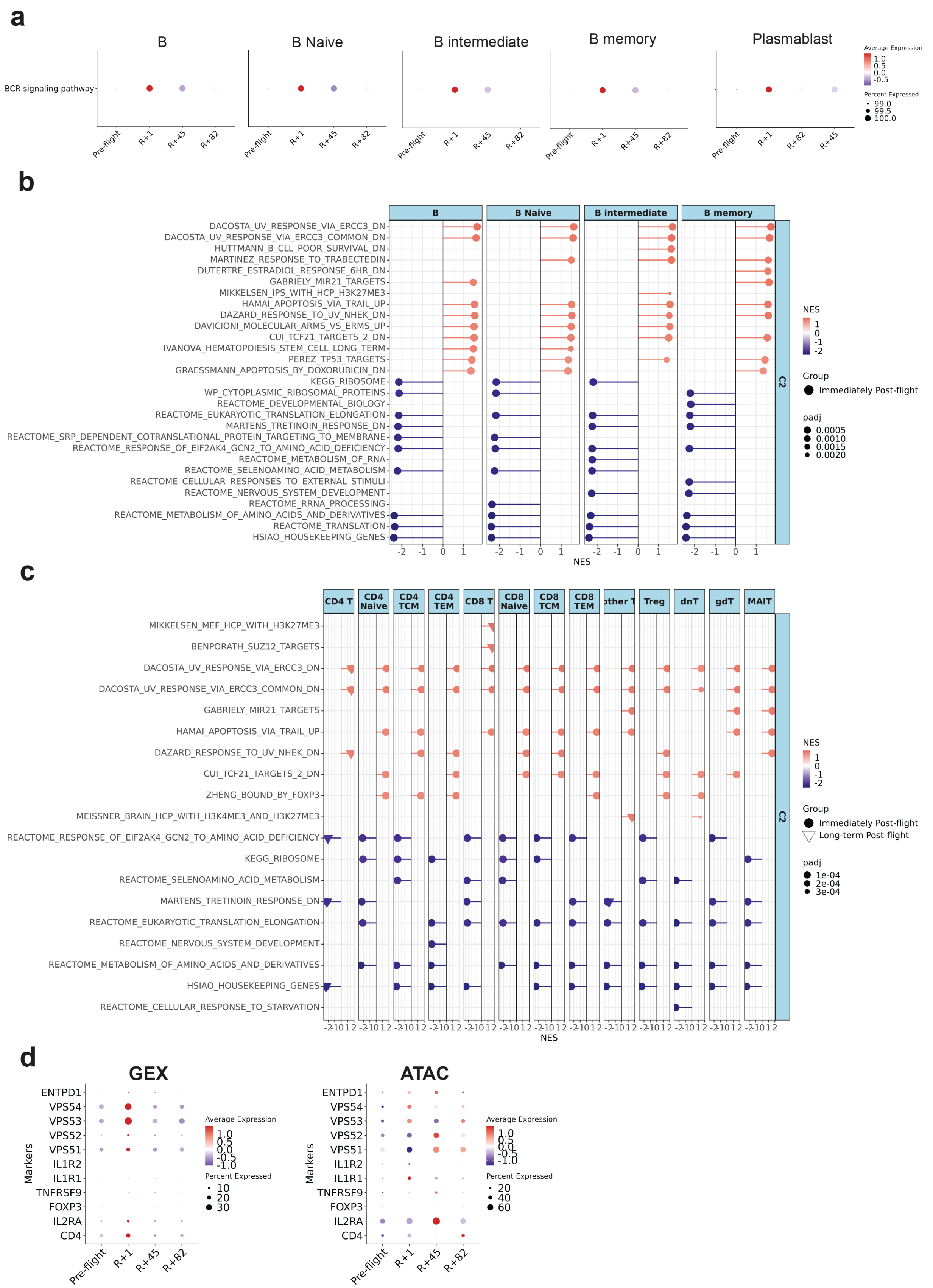
a
B
B Naive
B intermediate
B memory
Plasmablast
BCR signaling pathway
Pre-flight
R+1
R+45
R+82
Average Expression
1.0
0.5
0.0
-0.5
Percent Expressed
99.0
99.5
100.0
b
B
B Naive
B intermediate
B memory
DACOSTA_UV_RESPONSE_VIA_ERCC3_DN
DACOSTA_UV_RESPONSE_VIA_ERCC3_COMMON_DN
HUTTMANN_B_CLL_POOR_SURVIVAL_DN
MARTINEZ_RESPONSE_TO_TRABECTEDIN
DUTERTRE_ESTRADIOL_RESPONSE_6HR_DN
GABRIELY_MIR21_TARGETS
MIKKELSEN_IPS_WITH_HCP_H3K27ME3
HAMAI_APOPTOSIS_VIA_TRAIL_UP
DAZARD_RESPONSE_TO_UV_NHEK_DN
DAVICIONI_MOLECULAR_ARMS_VS_ERMS_UP
CUI_TCF21_TARGETS_2_DN
IVANOVA_HEMATOPOIESIS_STEM_CELL_LONG_TERM
PEREZ_TP53_TARGETS
GRAESSMANN_APOPTOSIS_BY_DOXORUBICIN_DN
KEGG_RIBOSOME
WP_CYTOPLASMIC_RIBOSOMAL_PROTEINS
REACTOME_DEVELOPMENTAL_BIOLOGY
REACTOME_EUKARYOTIC_TRANSLATION_ELONGATION
MARTENS_TRETINOIN_RESPONSE_DN
REACTOME_SRP_DEPENDENT_COTRANSLATIONAL_PROTEIN_TARGETING_TO_MEMBRANE
REACTOME_RESPONSE_OF_EIF2AK4_GCN2_TO_AMINO_ACID_DEFICIENCY
REACTOME_METABOLISM_OF_RNA
REACTOME_SELENOAMINO_ACID_METABOLISM
REACTOME_CELLULAR_RESPONSES_TO_EXTERNAL_STIMULI
REACTOME_NERVOUS_SYSTEM_DEVELOPMENT
REACTOME_RRNA_PROCESSING
REACTOME_METABOLISM_OF_AMINO_ACIDS_AND_DERIVATIVES
REACTOME_TRANSLATION
HSIAO_HOUSEKEEPING_GENES
C2
NES
Group
Immediately Post-flight
padj
0.0005
0.0010
0.0015
0.0020
c
CD4 T
CD4 Naive
CD4 TCM
CD4 TEM
CD8 T
CD8 Naive
CD8 TCM
CD8 TEM
other T
Treg
dnT
gdT
MAIT
MIKKELSEN_MEF_HCP_WITH_H3K27ME3
BENPORATH_SUZ12_TARGETS
DACOSTA_UV_RESPONSE_VIA_ERCC3_DN
DACOSTA_UV_RESPONSE_VIA_ERCC3_COMMON_DN
GABRIELY_MIR21_TARGETS
HAMAI_APOPTOSIS_VIA_TRAIL_UP
DAZARD_RESPONSE_TO_UV_NHEK_DN
CUI_TCF21_TARGETS_2_DN
ZHENG_BOUND_BY_FOXP3
MEISSNER_BRAIN_HCP_WITH_H3K4ME3_AND_H3K27ME3
REACTOME_RESPONSE_OF_EIF2AK4_GCN2_TO_AMINO_ACID_DEFICIENCY
KEGG_RIBOSOME
REACTOME_SELENOAMINO_ACID_METABOLISM
MARTENS_TRETINOIN_RESPONSE_DN
REACTOME_EUKARYOTIC_TRANSLATION_ELONGATION
REACTOME_NERVOUS_SYSTEM_DEVELOPMENT
REACTOME_METABOLISM_OF_AMINO_ACIDS_AND_DERIVATIVES
HSIAO_HOUSEKEEPING_GENES
REACTOME_CELLULAR_RESPONSE_TO_STARVATION
C2
NES
Group
Immediately Post-flight
Long-term Post-flight
padj
1e-04
2e-04
3e-04
d
GEX
ATAC
ENTPD1
VPS54
VPS53
VPS52
VPS51
IL1R2
IL1R1
TNFRSF9
FOXP3
IL2RA
CD4
Markers
Average Expression
Percent Expressed
10
20
30
40
60

Supplementary Figure 13

a, GSEA of PBMC and subpopulations from females and males at R+1 with the MSigDB hallmark (padj < 0.2). A one-sided permutation-based test to determine the significance of gene set enrichment, with raw p-values adjusted for multiple testing using the Benjamini-Hochberg procedure to control the false discovery rate (FDR). b, Activity scores of top enriched motifs from PBMC subpopulations separated by sex. Source data are provided as a Source Data file.

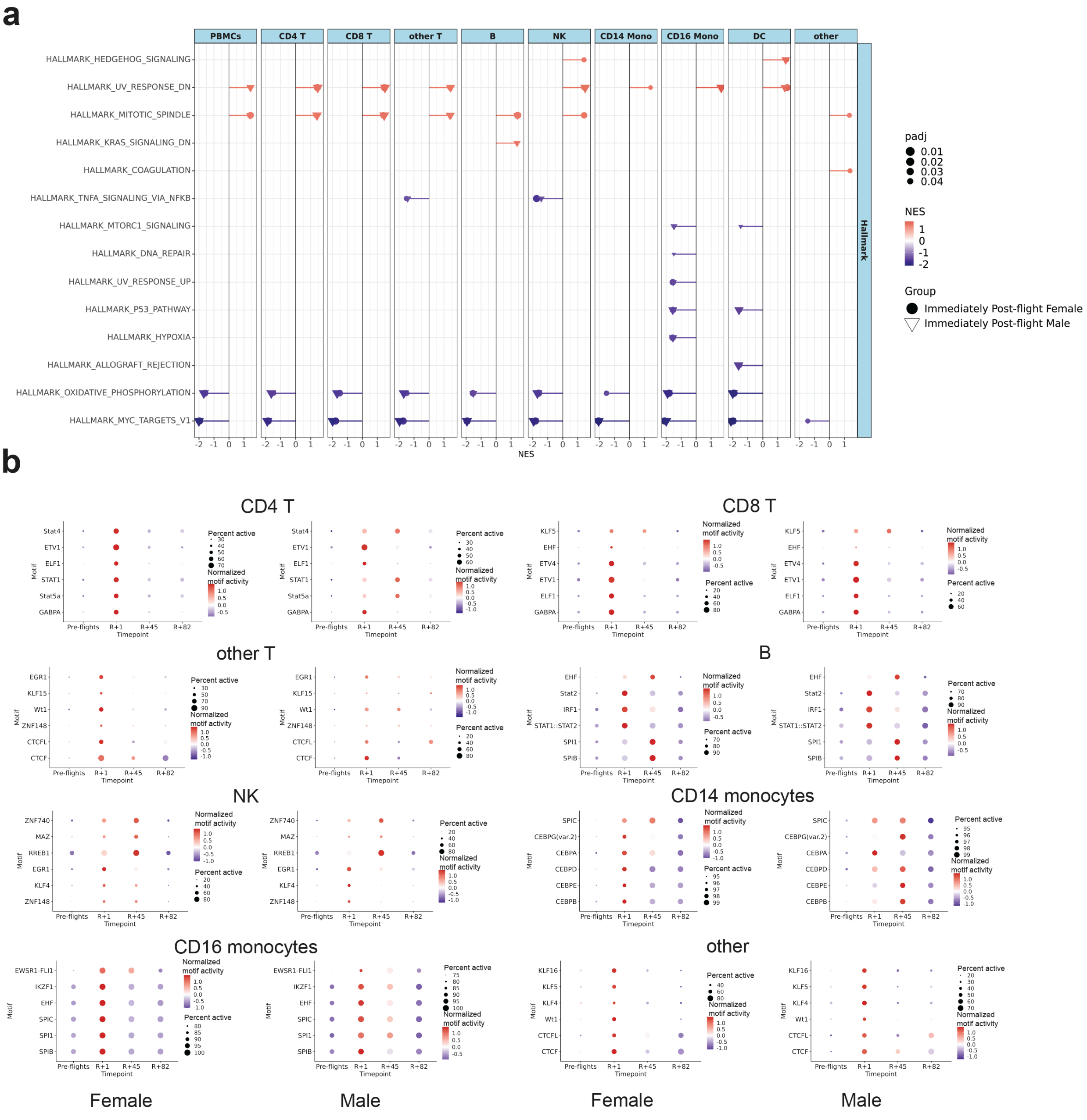

Supplementary Figure 14. Comparing association stability across taxonomic classifiers. a, Log-transformed associations on MetaPhlAn4-classified bacterial species and Phanta-classified viruses. Each point in the plot bodies represent a different bacterial species (top) or viral genus (bottom). The y-axes describe different human genes. For each cell type, we ranked genes with non-zero LASSO coefficients first by the number of Bonferroni < 0.2 findings, then by the total number of nominally associated (p-value < 0.05) microbial features (bacteria or viruses). We report up to ten human genes per sub-panel. b, This panel contains the same ranking and plotting strategy as panel a, except the associations were computed on CLR-transformed data. Lasso regression and the mixed effect linear regression approach were used for p-value estimation (two-sided). Bonferroni correction was used to adjust for multiple hypothesis testing. Source data are provided through the github link.

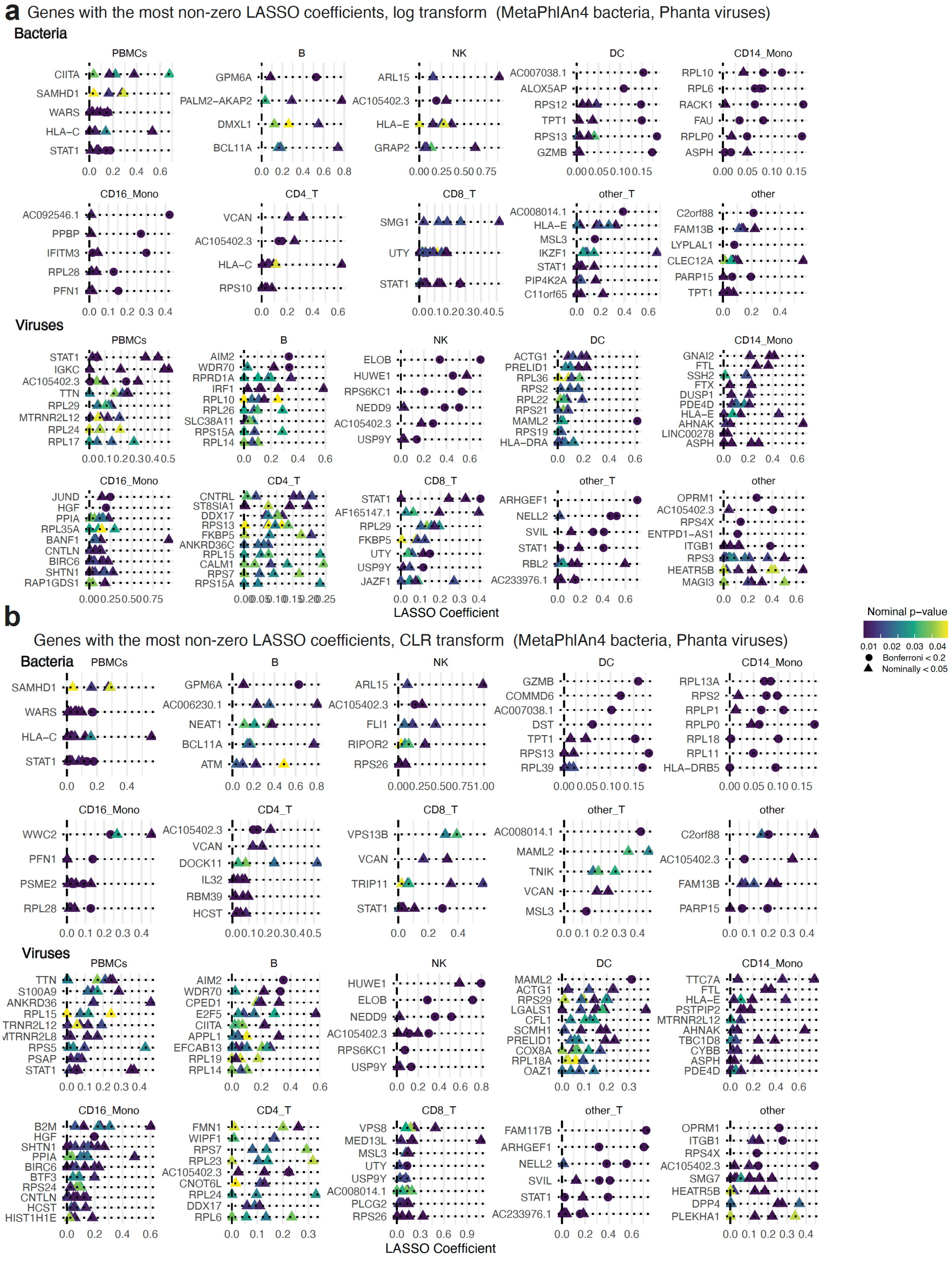

a Genes with the most non-zero LASSO coefficients, log transform (MetaPhlAn4 bacteria, Phanta viruses)
Bacteria
PBMCs
CIITA
SAMHD1
WARS
HLA−C
STAT1
B
GPM6A
PALM2−AKAP2
DMXL1
BCL11A
NK
ARL15
AC105402.3
HLA−E
GRAP2
DC
AC007038.1
ALOX5AP
RPS12
TPT1
RPS13
GZMB
CD14_Mono
RPL10
RPL6
RACK1
FAU
RPLP0
ASPH
CD16_Mono
AC092546.1
PPBP
IFITM3
RPL28
PFN1
CD4_T
VCAN
AC105402.3
HLA−C
RPS10
CD8_T
SMG1
UTY
STAT1
other_T
AC008014.1
HLA−E
MSL3
IKZF1
STAT1
PIP4K2A
C11orf65
other
C2orf88
FAM13B
LYPLAL1
CLEC12A
PARP15
TPT1
Viruses
PBMCs
STAT1
IGKC
AC105402.3
TTN
RPL29
MTRNR2L12
RPL24
RPL17
B
AIM2
WDR70
RPRD1A
IRF1
RPL10
RPL26
SLC38A11
RPS15A
RPL14
NK
ELOB
HUWE1
RPS6KC1
NEDD9
AC105402.3
USP9Y
DC
ACTG1
PRELID1
RPL36
RPS2
RPL22
RPS21
MAML2
RPS19
HLA−DRA
CD14_Mono
GNAI2
FTL
SSH2
FTX
DUSP1
PDE4D
HLA−E
AHNAK
LINC00278
ASPH
CD16_Mono
JUND
HGF
PPIA
RPL35A
BANF1
CNTLN
BIRC6
SHTN1
RAP1GDS1
CD4_T
CNTRL
ST8SIA1
DDX17
RPS13
FKBP5
ANKRD36C
RPL15
CALM1
RPS7
RPS15A
CD8_T
STAT1
AF165147.1
RPL29
FKBP5
UTY
USP9Y
JAZF1
other_T
ARHGEF1
NELL2
SVIL
STAT1
RBL2
AC233976.1
other
OPRM1
AC105402.3
RPS4X
ENTPD1−AS1
ITGB1
RPS3
HEATR5B
MAGI3
LASSO Coefficient
Nominal p−value
0.01 0.02 0.03 0.04
Bonferroni < 0.2
Nominally < 0.05
b
Genes with the most non-zero LASSO coefficients, CLR transform (MetaPhlAn4 bacteria, Phanta viruses)
Bacteria
PBMCs
SAMHD1
WARS
HLA−C
STAT1
B
GPM6A
AC006230.1
NEAT1
BCL11A
ATM
NK
ARL15
AC105402.3
FLI1
RIPOR2
RPS26
DC
GZMB
COMMD6
AC007038.1
DST
TPT1
RPS13
RPL39
CD14_Mono
RPL13A
RPS2
RPLP1
RPLP0
RPL18
RPL11
HLA−DRB5
CD16_Mono
WWC2
PFN1
PSME2
RPL28
CD4_T
AC105402.3
VCAN
DOCK11
IL32
RBM39
HCST
CD8_T
VPS13B
VCAN
TRIP11
STAT1
other_T
AC008014.1
MAML2
TNIK
VCAN
MSL3
other
C2orf88
AC105402.3
FAM13B
PARP15
Viruses
PBMCs
TTN
S100A9
ANKRD36
RPL15
TRNR2L12
MTRNR2L8
RPS5
PSAP
STAT1
B
AIM2
WDR70
CPED1
E2F5
CIITA
APPL1
EFCAB13
RPL19
RPL14
NK
HUWE1
ELOB
NEDD9
AC105402.3
RPS6KC1
USP9Y
DC
MAML2
ACTG1
RPS29
LGALS1
CFL1
SCMH1
PRELID1
COX8A
RPL18A
OAZ1
CD14_Mono
TTC7A
FTL
HLA−E
PSTPIP2
MTRNR2L12
AHNAK
TBC1D8
CYBB
ASPH
PDE4D
CD16_Mono
B2M
HGF
SHTN1
PPIA
BIRC6
BTF3
RPS24
CNTLN
HCST
HIST1H1E
CD4_T
FMN1
WIPF1
RPS7
RPL23
AC105402.3
CNOT6L
RPL24
DDX17
RPL6
CD8_T
VPS8
MED13L
MSL3
UTY
USP9Y
AC008014.1
PLCG2
RPS26
other_T
FAM117B
ARHGEF1
NELL2
SVIL
STAT1
AC233976.1
other
OPRM1
ITGB1
RPS4X
AC105402.3
SMG7
HEATR5B
DPP4
PLEKHA1
LASSO Coefficient

Supplementary Figure 15. Alternative methods for exploring microbiome-immune associations. a, We computed LASSO and mixed modeling associations using Center-Log-Ratio instead of log-transformed data. This was done to compare that our results were stable under multiple compositional data analysis methods. The three bars in each sub-panel correspond to the number of associations in the “real” (log-transformed) data versus CLR data and the overlap therein at different stringency levels in controlling for false positives. b, The human genes, per cell type, with the greatest number of microbial associations that themselves had low or Bonferroni-significant p-values. Each point in the plot bodies represents a different bacterial species (top) or viral genus (bottom). For each cell type, we ranked genes with non-zero LASSO coefficients first by the number of Bonferroni < 0.2 findings, then by the total number of nominally associated (p-value < 0.05) microbial features (bacteria or viruses). We report up to ten human genes per sub-panel. Lasso regression and the mixed effect linear regression approach were used for p-value estimation (two-sided). Bonferroni correction was used to adjust for multiple hypothesis testing. Source data are provided through the github link.

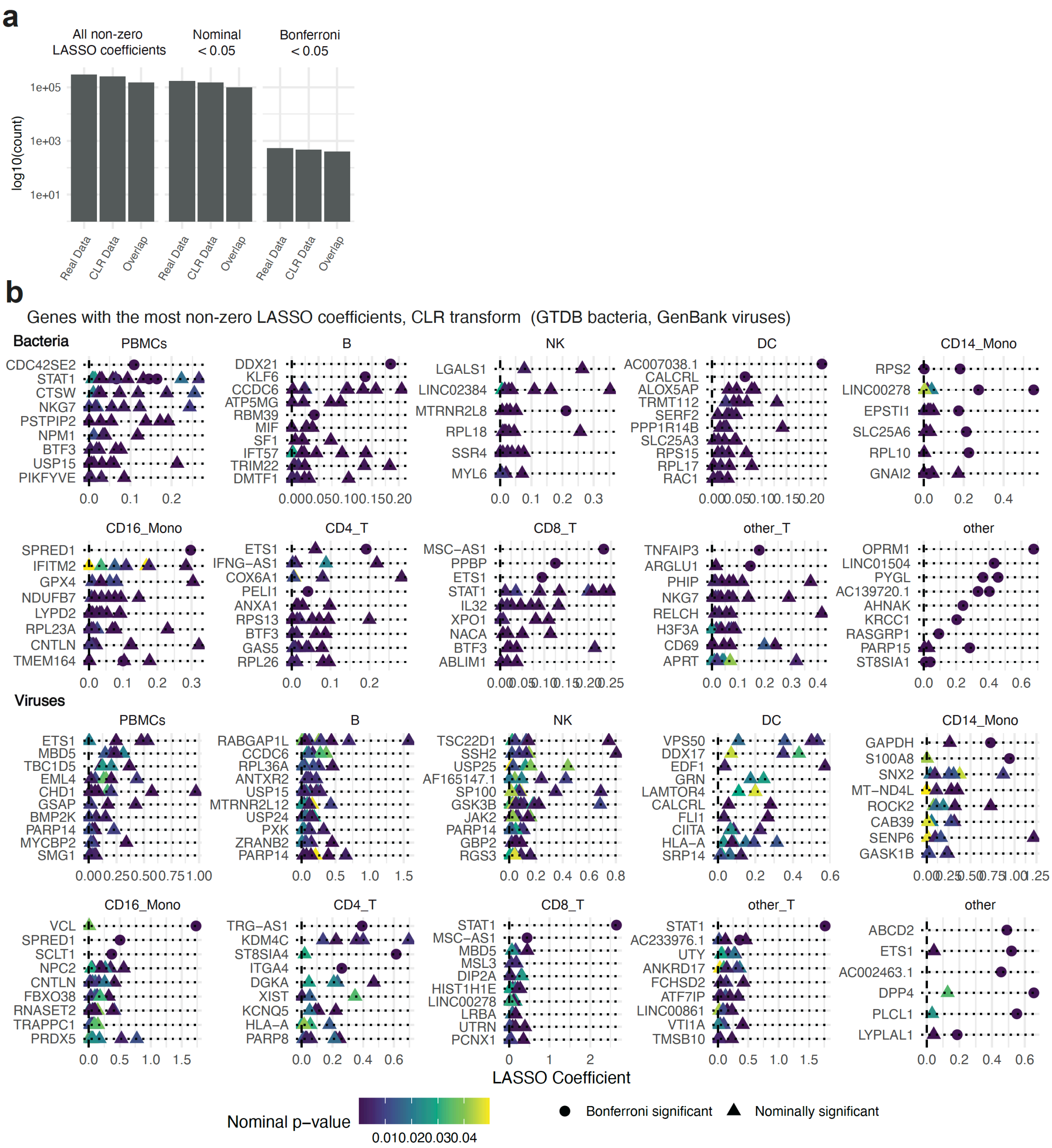
a
All non-zero LASSO coefficients
Nominal < 0.05
Bonferroni < 0.05
log10(count)
1e+05
1e+03
1e+01
Real Data
CLR Data
Overlap
b
Genes with the most non-zero LASSO coefficients, CLR transform (GTDB bacteria, GenBank viruses)
Bacteria
Viruses
PBMCs
B
NK
DC
CD14_Mono
CD16_Mono
CD4_T
CD8_T
other_T
other
LASSO Coefficient
Nominal p−value
0.01 0.02 0.03 0.04
Bonferroni significant
Nominally significant
