# Supplementary material for: Single-cell multi-ome and immune profiles of the Inspiration4 crew reveal conserved, cell-type, and sex-specific responses to spaceflight
Source: Nat Commun. 2024 Jun 11;15:4954. doi: 10.1038/s41467-024-49211-2 (PMC11166952; doi:10.1038/s41467-024-49211-2)
Supplement: Supplementary file 3 — Description of Additional Supplementary Files [file 41467_2024_49211_MOESM3_ESM.pdf]

Description of Additional Supplementary Files

Supplementary Data 1

Significantly enriched pathways of over-representation analysis of the spaceflight signatures of the I4 astronauts with GO-BP pathway. Filtered with adjusted p-value < 0.05. A hypergeometric test to assess the significance of enriched gene sets, with raw p-values adjusted for multiple testing using the Benjamini-Hochberg procedure to control the false discovery rate (FDR).

Supplementary Data 2

Pathways enriched in I4 immune cell female and male shared DEGs at R+1 from Ingenuity Pathway Analysis (IPA). A right-tailed Fisher's exact test to determine the significance of pathway enrichment, with raw p-values adjusted for multiple testing using the Benjamini-Hochberg procedure to control the false discovery rate (FDR).

Supplementary Data 3

Enriched motif of the I4 immune cells. The list of the enriched motifs at R+1 from the I4 immune cells. A one-sided hypergeometric test to identify significantly enriched motifs, with raw p-values adjusted for multiple testing using the Bonferroni correction to control the family-wise error rate (FWER).

Supplementary Data 4

ssGSEA output of the immune cells.

Supplementary Data 5

DEGs identified from the meta-analysis of the GeneLab database. A Wald test to identify differentially expressed genes, with raw p-values adjusted for multiple testing using the Benjamini-Hochberg procedure to control the false discovery rate (FDR).

Supplementary Data 6

Significantly enriched pathways of over-representation analysis of the spaceflight signatures of mice with GO-BP pathway. Filtered with adjusted p-value < 0.05. A hypergeometric test to assess the significance of enriched gene sets, with raw p-values adjusted for multiple testing using the Benjamini-Hochberg procedure to control the false discovery rate (FDR).

Supplementary Data 7

List of the potential drugs and compounds for countermeasures. Potential drug and compounds (padj < 0.05) derived from DEGs of I4 immune cells. The test is one sided Fisher's exact test and Benjamini-Hochberg (FDR) method was used for multiple comparison adjustment.

Supplementary Data 8

Significantly enriched pathways of over-representation analysis of the I4 immune cell DEGs with KEGG pathway. Filtered with adjusted p-value < 0.05. A hypergeometric test to assess the significance of enriched gene sets, with raw p-values adjusted for multiple testing using the Benjamini-Hochberg procedure to control the false discovery rate (FDR).

Supplementary Data 9

Pathways enriched in I4 immune cell female and male unique DEGs at R+1 from Ingenuity pathway analysis (IPA). A right-tailed Fisher's exact test to determine the significance of pathway enrichment, with raw p-values adjusted for multiple testing using the Benjamini-Hochberg procedure to control the false discovery rate (FDR).

Supplementary Data 10

Fold change, p-value, and adjusted p-value of CD and HLA genes in the I4 female and male immune cells. Wilcoxon rank-sum test to identify differentially expressed genes between clusters, with raw p-values adjusted for multiple testing using the Bonferroni correction to control the family-wise error rate (FWER).

Supplementary Data 11

Enriched motifs of the I4 female and immune cells. The list of the enriched motifs at R+1 from the I4 female and male immune cells. A one-sided hypergeometric test to identify significantly enriched motifs, with raw p-values adjusted for multiple testing using the Bonferroni correction to control the family-wise error rate (FWER).

Supplementary Data 12

Biochemical profiles of astronauts separated by sex. Two-way ANOVA with a post hoc Bonferroni t-test.

Supplementary Data 13

Microbiome immune associations. The output from the lasso regressions between microbiome abundance and immune cell DEGs. Lasso regression and the mixed effect linear regression approach were used for p-value estimation (two-sided). Bonferroni correction was used to adjust for multiple hypothesis testing.

Supplementary Data 14

Significantly enriched pathways of overrepresentation analysis of the I4 immune cell DEGs with GO-BP pathway. Filtered with adjusted p-value < 0.05. A hypergeometric test to assess the significance of enriched gene sets, with raw p-values adjusted for multiple testing using the Benjamini-Hochberg procedure to control the false discovery rate (FDR).

Supplementary Data 15

DEGs and the overlapped GSEA pathway across studies. The fgsea analysis employs a one-sided permutation-based test to determine the significance of gene set enrichment, with raw p-values adjusted for multiple testing using the Benjamini-Hochberg procedure to control the false discovery rate (FDR).
