# Supplementary material for: Single-cell multi-ome and immune profiles of the Inspiration4 crew reveal conserved, cell-type, and sex-specific responses to spaceflight
Source: Nat Commun. 2024 Jun 11;15:4954. doi: 10.1038/s41467-024-49211-2 (PMC11166952; doi:10.1038/s41467-024-49211-2)
Supplement: Supplementary file 5 — Reporting Summary [file 41467_2024_49211_MOESM5_ESM.pdf]

nature portfolio

Corresponding author(s): Christopher E. Mason

Last updated by author(s): Apr 23, 2024

# Reporting Summary

Nature Portfolio wishes to improve the reproducibility of the work that we publish. This form provides structure for consistency and transparency in reporting. For further information on Nature Portfolio policies, see our Editorial Policies and the Editorial Policy Checklist.

## Statistics

For all statistical analyses, confirm that the following items are present in the figure legend, table legend, main text, or Methods section.

| n/a | Confirmed | |
|---|---|---|
| ☐ | ☒ | The exact sample size (*n*) for each experimental group/condition, given as a discrete number and unit of measurement |
| ☐ | ☒ | A statement on whether measurements were taken from distinct samples or whether the same sample was measured repeatedly |
| ☐ | ☒ | The statistical test(s) used AND whether they are one- or two-sided<br>*Only common tests should be described solely by name; describe more complex techniques in the Methods section.* |
| ☐ | ☒ | A description of all covariates tested |
| ☐ | ☒ | A description of any assumptions or corrections, such as tests of normality and adjustment for multiple comparisons |
| ☐ | ☒ | A full description of the statistical parameters including central tendency (e.g. means) or other basic estimates (e.g. regression coefficient) AND variation (e.g. standard deviation) or associated estimates of uncertainty (e.g. confidence intervals) |
| ☐ | ☒ | For null hypothesis testing, the test statistic (e.g. *F*, *t*, *r*) with confidence intervals, effect sizes, degrees of freedom and *P* value noted<br>*Give P values as exact values whenever suitable.* |
| ☒ | ☐ | For Bayesian analysis, information on the choice of priors and Markov chain Monte Carlo settings |
| ☒ | ☐ | For hierarchical and complex designs, identification of the appropriate level for tests and full reporting of outcomes |
| ☐ | ☒ | Estimates of effect sizes (e.g. Cohen's *d*, Pearson's *r*), indicating how they were calculated |

*Our web collection on statistics for biologists contains articles on many of the points above.*

## Software and code

Policy information about availability of computer code

| | |
|---|---|
| Data collection | N/A |
| Data analysis | Cell Ranger arc (v2.0.0)<br>Cell Ranger (v6.1.1)<br>Azimuth (v0.3.2)<br>Seurat (v4.2.0)<br>chromVAR (v1.16.0)<br>VGenes (vPre-release3)<br>DESeq2 (cfRNA: v1.34.0. Mouse GeneLab: v.1.38.3)<br>ComplexHeatmap (v.2.15.1)<br>EnhancedVolcano (v.1.16.0)<br>fgsea (v1.22)<br>GSVA (v1.42.0)<br>msigdbr (v7.4.1)<br>gprofiler (v.e111_eg58_p18_30541362)<br>GeneOverlap (v.1.34.0)<br>pheatmap (v1.0)<br>bbtools (v38.92)<br>XTree (v0.92i)<br>Kraken2 (v2.1.2)<br>bracken (v2.6.2) |

vegan (v2.6.2)
MetaSPAdes (v3.14.3)
MetaQUAST (v5.0.2)
Bowtie2 (v2.2.3)
samtools (v1.0, 1.9)
MMseqs2 (v13.4511)
Diamond (v2.0.14)
Snakemake workflow management system (v7.7.0)
STAR (v2.7.0f)
UMI tools (v1.1.2)
featureCount (v2.0.0)
BayesPrism (v2.0)

For manuscripts utilizing custom algorithms or software that are central to the research but not yet described in published literature, software must be made available to editors and reviewers. We strongly encourage code deposition in a community repository (e.g. GitHub). See the Nature Portfolio guidelines for submitting code & software for further information.

## Data

Policy information about availability of data

All manuscripts must include a data availability statement. This statement should provide the following information, where applicable:

- Accession codes, unique identifiers, or web links for publicly available datasets
- A description of any restrictions on data availability
- For clinical datasets or third party data, please ensure that the statement adheres to our policy

Datasets have been uploaded to two data repositories: the NASA Open Science Data Repositories (OSDR; osdr.nasa.gov; comprised of GeneLab9 and the Ames Life Sciences Data Archive [ALSDA]), and the TrialX database. Identifiers for publicly downloadable datasets in the OSDR are documented below. Select data can be visualized online through the SOMA Data Explorer: https://soma.weill.cornell.edu/#main. Also, the single-cell data can be visualized online through Inspiration4 Multiome Data Explorer: https://epigenetics.weill.cornell.edu/apps/I4_Multiome/. All code used to generate Figures and analyses from this project is available at https://github.com/eliah-o/inspiration4-omics.

## Human research participants

Policy information about studies involving human research participants and Sex and Gender in Research.

| | |
|---|---|
| Reporting on sex and gender | Yes, sex information was collected and is stated in Figure 3. However, all analyses are performed with the crew as a single cohort. |
| Population characteristics | The crew member composition was of two races and ages ranged from 29-51. |
| Recruitment | Participants were recruited by SpaceX and mission commander Jared Isaacman. |
| Ethics oversight | All subjects were consented at an informed consent briefing (ICB) at SpaceX (Hawthorne, CA), and samples were collected and processed under the approval of the Institutional Review Board (IRB) at Weill Cornell Medicine, under Protocol 21-05023569. All crew members have consented for data and sample sharing. |

Note that full information on the approval of the study protocol must also be provided in the manuscript.

# Field-specific reporting

Please select the one below that is the best fit for your research. If you are not sure, read the appropriate sections before making your selection.

☒ Life sciences ☐ Behavioural & social sciences ☐ Ecological, evolutionary & environmental sciences

For a reference copy of the document with all sections, see nature.com/documents/nr-reporting-summary-flat.pdf

# Life sciences study design

All studies must disclose on these points even when the disclosure is negative.

| | |
|---|---|
| Sample size | The entire Inspiration4 crew was profiled, which was limited by the size of the Dragon capsule (n=4). |
| Data exclusions | No data has been excluded. |
| Replication | Replication tests are difficult as mission parameters cannot be repeated. Where possible, data validation was performed via qPCR to validate transcriptomic findings. Experiments involving human material (including the qPCR), were only performed once due to limited immune cell availability. |

| | |
|---|---|
| Randomization | This is not relevant to the study as we were profiling the entire crew longitudinally (at different timepoints pre-flight and post-flight) and therefore it was not possible to randomize the collection. |
| Blinding | Blinding was not possible because all subjects were astronauts in the same crew. |

# Reporting for specific materials, systems and methods

We require information from authors about some types of materials, experimental systems and methods used in many studies. Here, indicate whether each material, system or method listed is relevant to your study. If you are not sure if a list item applies to your research, read the appropriate section before selecting a response.

## Materials & experimental systems

| n/a | Involved in the study | |
|---|---|---|
| ☐ | ☒ | Antibodies |
| ☒ | ☐ | Eukaryotic cell lines |
| ☒ | ☐ | Palaeontology and archaeology |
| ☒ | ☐ | Animals and other organisms |
| ☒ | ☐ | Clinical data |
| ☒ | ☐ | Dual use research of concern |

## Methods

| n/a | Involved in the study | |
|---|---|---|
| ☒ | ☐ | ChIP-seq |
| ☐ | ☒ | Flow cytometry |
| ☒ | ☐ | MRI-based neuroimaging |

## Antibodies

| | |
|---|---|
| Antibodies used | CD3 (T cells) (BD, cat no.555342)<br>CD19 (B cells) (Biolegend, cat no.302207)<br>CD14 (Monocyte) (BD, cat no.563420)<br>CD56 (for NK cells) (BD, cat no.564058) |
| Validation | The cells were resuspended and incubated with monocyte blocking solution (Biolegend, cat no.426102) and Fc Receptor Blocking Solution (Biolegend, cat no.422301) for 20 mins.<br><br>Information from the manufacturer:<br>CD56 (5 ul per million cells in 100 ul): This reagent has been pre-diluted for use at the recommended Volume per Test. We typically use 1 × 10^6 cells in a 100-µl experimental sample (a test).<br>CD3 (20 ul per million cells in 100 ul): This reagent has been pre-diluted for use at the recommended Volume per Test. We typically use 1 × 10^6 cells in a 100-µl experimental sample (a test).<br>CD14 (5 ul per million cells in 100 ul): This reagent has been pre-diluted for use at the recommended Volume per Test. We typically use 1 × 10^6 cells in a 100-µl experimental sample (a test).<br>CD19 (5 ul per million in 100 ul): For flow cytometric staining using the µg size, the suggested use of this reagent is ≤0.25 µg per million cells in 100 µl volume. It is recommended that the reagent be titrated for optimal performance for each application. For flow cytometric staining using the test sizes, the suggested use of this reagent is 5 µl per million cells in 100 µl staining volume or 5 µl per 100 µl of whole blood. |

## Flow Cytometry

### Plots

Confirm that:

☒ The axis labels state the marker and fluorochrome used (e.g. CD4-FITC).

☒ The axis scales are clearly visible. Include numbers along axes only for bottom left plot of group (a 'group' is an analysis of identical markers).

☒ All plots are contour plots with outliers or pseudocolor plots.

☒ A numerical value for number of cells or percentage (with statistics) is provided.

### Methodology

| | |
|---|---|
| Sample preparation | The granulocyte-depleted peripheral blood samples were subjected to fluorescence-activated cell sorting to sort out different subsets of immune cells. Briefly, the frozen samples were thawed, and washed in cell staining buffer (Biolegend, cat no.420201). The cells were resuspended and incubated with monocyte blocking solution (Biolegend, cat no.426102) and Fc Receptor Blocking Solution (Biolegend, cat no.422301) for 20 mins, followed by incubation with CD3 (T cells) (BD, cat no.555342), CD19 (B cells) (Biolegend, cat no.302207), CD14 (Monocyte) (BD, cat no.563420), and CD56 (for NK cells) (BD, cat no.564058) for 30 minutes, washed twice in the cell staining buffer and resuspended in the same buffer. Right before sorting DAPI was added to eliminate dead cells during sorting. |
| Instrument | BD FACSAris Fusion. Serial #: 127896. Model #: 25172 |

Software

BD FACSDiva™ software

Cell population abundance

| | B | T | NK | Mono |
|---|---|---|---|---|
| C001_L-44 | 13,811 | 202,316 | 75,105 | 28,333 |
| C001_R+1 | 7,868 | 67,863 | 18,412 | 7,680 |
| C001_R+82 | 24,615 | 184,790 | 42,614 | 20,720 |
| C002_L-44 | 47,067 | 806,810 | 48,108 | 58,376 |
| C002_R+1 | 6,508 | 53,583 | 7,527 | 12,484 |
| C002_R+82 | 54,712 | 442,954 | 46,448 | 35,244 |
| C003_L-44 | 66,392 | 360,694 | 66,942 | 53,040 |
| C003_R+1 | 3,153 | 55,281 | 5,034 | 134 |
| C003_R+82 | 101,015 | 765,104 | 68,155 | 85,385 |
| C004_L-44 | 60,718 | 394,584 | 66,840 | 67,139 |
| C004_R+1 | 55,157 | 117,920 | 19,661 | 20,398 |
| C004_R+82 | 85,358 | 757,223 | 101,530 | 141,895 |

Gating strategy

The gating strategy was shown in the Extended Data Fig. 3d.

☒ Tick this box to confirm that a figure exemplifying the gating strategy is provided in the Supplementary Information.
