# Supplementary material for: Single-cell multi-ome and immune profiles of the Inspiration4 crew reveal conserved, cell-type, and sex-specific responses to spaceflight
Source: Nat Commun. 2024 Jun 11;15:4954. doi: 10.1038/s41467-024-49211-2 (PMC11166952; doi:10.1038/s41467-024-49211-2)
Supplement: Supplementary file 2 — Peer Review File [file 41467_2024_49211_MOESM2_ESM.pdf]

nature portfolio

Peer Review File

Single-cell multi-ome and immune profiles of the Inspiration4 crew reveal conserved, cell-type, and sex-specific responses to spaceflight

Open Access This file is licensed under a Creative Commons Attribution 4.0 International License, which permits use, sharing, adaptation, distribution and reproduction in any medium or format, as long as you give appropriate credit to the original author(s) and the source, provide a link to the Creative Commons license, and indicate if changes were made. In the cases where the authors are anonymous, such as is the case for the reports of anonymous peer reviewers, author attribution should be to 'Anonymous Referee' followed by a clear attribution to the source work. The images or other third party material in this file are included in the article's Creative Commons license, unless indicated otherwise in a credit line to the material. If material is not included in the article's Creative Commons license and your intended use is not permitted by statutory regulation or exceeds the permitted use, you will need to obtain permission directly from the copyright holder. To view a copy of this license, visit http://creativecommons.org/licenses/by/4.0/.

Editorial Note: This manuscript has been previously reviewed at another journal. This document only contains reviewer comments and rebuttal letters for versions considered at *Nature Communications*.

REVIEWERS' COMMENTS

Reviewer #1 (Remarks to the Author):

I have carefullly considered the manuscript and the response to the reviewers document, with a particular focus on reviewer number 4 requests and authors responses to these comment.
In my opinion I think the authors comprehensively addressed the requests of reviewer 4, particularly around the issues of statistics and data analysis. The authors have integrated their dataset with almost every available datasets (including other resources from the same initiative), correctly performed statistical analyses and more importantly, acknowledged the limitations of the current study. Overall, I do not think the authors have left any stone unturned and the findings are as robust as possibly can considering the experimental design (which is not trivial considering the inherent constrains in these types of experiments).
